# Recommendations for the optimization of student led free vision screening programs

**DOI:** 10.1186/s12909-024-06396-w

**Published:** 2024-12-18

**Authors:** Nirupama Devanathan, Melanie Scheive, Amrish Selvam, Baraa S. Nawash, Alec Murphy, McKenna Morrow, Shruti Anant, Jessica S. Kruger, Chi-Wah Rudy Yung, Thomas V. Johnson

**Affiliations:** 1 Department of Ophthalmology, Eugene and Marilyn Glick Eye Institute, Indiana University School of Medicine, Indianapolis, IN USA; 2 Department of Ophthalmology, University of Pittsburgh School of Medicine, Pittsburgh, PA USA; 3 Department of Ophthalmology, University of Cincinnati College of Medicine, Cincinnati, OH USA; 4 Department of Ophthalmology and Visual Sciences, School of Medicine, Medical College of Wisconsin, Milwaukee, WI USA; 5 Wilmer Eye Institute, Johns Hopkins University School of Medicine, Baltimore, MD USA; 6 Department of Community Health and Health Behavior, University at Buffalo School of Public Health and Health Professions, Buffalo, NY USA

**Keywords:** Vision screening, SWOT, Student-run free clinics, Preventative health

## Abstract

**Purpose:**

To report the summary characteristics of operational models associated with Student Led Free Vision Screening Programs (SLFVSP) and to identify opportunities for program optimization.

**Methods:**

An 81-question mixed methods survey was distributed to SLFVSP leaders nationwide and Medical Student Educators within the American University Ophthalmology Professors (AUPO) Association. Survey responses were analyzed using Mann Whitney U and Fisher’s Exact tests. Themes considering the assets and liabilities of SLFVSPs were summarized using self-reported qualitative data from survey responses. Qualitative and quantitative themes considering were then synthesized into a Strengths, Weaknesses, Opportunities, & Threats (SWOT) analysis for a collective appraisal of SLFVSP operations. Finally, drivers were identified to generate change ideas to improve SLFVP operations through a collaborative, quality improvement model.

**Results:**

A total of 16 survey responses were included from programs operational for a median of 6 years. Most respondent programs (*n* = 9) reported year-long operations; no preference between weekday (*n* = 8) and weekend (*n* = 7) screening activities was identified. Programs obtained funding from a diverse array of internal and external sources. There was no significant difference in wait time for scheduled appointments compared to a walk-in strategy; overall door-to-door visit times ranged from 15 min to 120 min. Screenings were held in several locations, most commonly in Federally Qualified Health Centers (*n* = 8) and religious centers (*n* = 6). Most screening event volunteers were first- and second-year medical students. The qualitative thematic analysis demonstrated that the most commonly self-reported asset was improving access to scarce vision screening services (*n* = 7) while the most commonly self-reported liability was difficulty recruiting faculty and/or resident for oversight (*n* = 5). The SWOT analysis revealed while the participant SLFVSPs were bolstered by site experience, community and corporate partnerships for glasses and space to hold vision screening, and institutional support from academic ophthalmology departments, limitations included difficulty with recruitment, space limitations, and poor follow-up care infrastructure.

**Conclusion:**

Collaborative standardization of SLFVSP operations can promote targeted staff training, organizational stewardship, and consensus building to ensure SLFVSP can offer sustainable vision screening programs that build vision equity at the community level.

**Supplementary Information:**

The online version contains supplementary material available at 10.1186/s12909-024-06396-w.

## Background

Although the U.S. Preventative Services Task Force (USPTF) cites insufficient evidence to support recommending widespread, routine vision screening in adults, especially due to limitations with adequate follow-up care [1], studies demonstrate that the underutilization of eye care by socioeconomically and marginalized populations results in indisputable harms from vision-threatening illness [2–4]. A lack of insurance and/or employment impedes access to routine eye care at intervals recommended by the American Academy of Ophthalmology. Community vision screening programs can help identify conditions like glaucoma and diabetic retinopathy that if left untreated, have devastating visual consequences [5–7]. One subset of community vision programs, Student Led Free Vision Screening Programs (SLFVSPs), offers free vision screening services to the public to bridge gaps in access while also supporting medical education to enhance ophthalmologic knowledge and promote engagement in community volunteer experiences [8, 9].

Building on the work of Okaka et al. [10], who surveyed characteristics of SLFVSPs associated with *primary care* student-run free clinics (SRFCs), the Consortium of Student Led Eye Clinics (CSLEC) launched a comprehensive survey to assess SLFVSPs nationwide, regardless of their affiliation with an SRFC. The CSLEC consists of medical students, residents, and university faculty involved with SLFVSPs throughout the United States seeking to improve access to quality eye care for underserved populations. We have previously reported on the service delivery capabilities and limitations of such programs, by delineating the extent by which SLFVSPs offer refractive, anterior segment, and fundus exams, and by describing the relative success rates of different follow-up care coordination protocols [11]. 

The primary aim of this report is to propose data-driven, experience-based recommendations regarding volunteer education, funding, and clinical operations based on aggregate quantitative and qualitative responses from the survey. This study expands on our initial CSLEC study [11] detailing the services, common diagnoses, and follow up protocols of SLFVSPs by analyzing the survey questions related to the volunteer, funding source, and operational aspects of these clinics.

A Strengths, Weaknesses, Opportunities, & Threats (SWOT) analysis was conducted to evaluate intrinsic and extrinsic characteristics associated with SLFVSP assets and liabilities. The SWOT framework is a useful element of strategic planning [9], and was developed to appraise the current state of SLFVSPs. While SWOT analyses in corporate and non-profit settings may inform stewardship and strategic planning for individual organizations, recent applications of this analysis method to the biomedical field have helped better characterize and optimize large entities that require the participation of numerous stakeholders [12, 13]. As a case in point, Sebel et al. [14], implemented SWOT to evaluate urology residencies, despite necessarily representing over one hundred individual programs. We employed SWOT analysis to answer the following question: “How can widespread adoption of a collaborative framework, like the CSLEC, help individual programs deliver optimal care”?

## Methods

An 81-item, Google form survey was administered from October 1, 2022, to February 24, 2023. The approach to survey construction and solicitation of participation was determined by a multi-institutional team within the CSLEC, including medical students and ophthalmology faculty. Institutional review board approval (IRB) was obtained at Indiana University School of Medicine, University of Pittsburgh School of Medicine, University of Cincinnati College of Medicine, Medical College of Wisconsin, and Johns Hopkins University School of Medicine. The survey included questions in several formats (open-ended, multiple choice, and yes/no) as detailed in the Supplement. Eligible survey participants were contacted directly through email. Invited participants included CSLEC members, ophthalmology faculty members of the Association for University Professors of Ophthalmology’s Medical Student Educators listserv, SLFVSP leaders as identified through web searches of 42 institutions, and faculty or students identified through communication networks among CSLEC ophthalmology faculty. Four rounds of outreach were made using our available lists. To ensure that only one survey response per SLFVSP was included, identifiable data was collected through a secure, online form; completion of the survey constituted informed consent for participation.

Quantitative data underwent systematic analysis, including the tabulation of responses from each participating program. The mean and standard deviation were calculated for quantitative responses, except for the total operational times, for which the median and interquartile range were calculated. Sub-group analyses were conducted using Mann Whitney U and Fisher’s Exact tests, accounting for a non-normal distribution and unequal variances among sub-groups. To ensure precision of outcomes, all data was cross verified by at least two members of the research team. Qualitative data was reviewed using a thematic analysis; key phrases and terms for open-ended questions were extracted. This data was first coded by one researcher (N.D.) and those codes were confirmed by a separate reviewer (J.S.K.) to ensure the themes agreed. Any themes that were not in agreement were discussed between the two researchers until a consensus was reached.

After aggregating themes into self-reported assets and liabilities, the quantitative responses were considered to further substantiate claims that were made qualitatively. The SWOT analysis was conducted by iteratively separating the characteristics which were intrinsic to a specific SLFVSP (Strengths and Weaknesses) from those that were extrinsic to the operation of a SLFVSP (Opportunities and Threats). In addition to considering characteristics derived from the survey response, a literature search was conducted to identify if similar traits were also identified in other student run, free clinics, given the smaller than expected sample size. If the literature provided additional characteristics for consideration in the SWOT framework, they were also included. Recognizing the limited sample size, care was taken to ensure quantitative data, qualitative data, and information from a general literature search corroborated with one another.

A driver diagram, a Quality Improvement (QI) framework, was then applied to consider how a collaborative model could be equipped to specifically address these weaknesses and threats that appeared to exist as common issues for individual clinics. Another iterative process was conducted to identify primary drivers that could summarize areas for collaborative problem solving, with secondary drivers and change ideas derived from the granularity of the survey responses. These in turn, shaped recommendations for the CSLEC as well as for individual SLFVSPs.

## Results

Responses from 16 SLFVSPs (15 institutions) were included in the final analysis from an original group of 23 responses. Four responses were excluded due to < 5% completion of questions, one due to duplicate entry, and two due to clinics not yet being operational [11]. One institution had two independent SLFVSPs, so these clinics were analyzed separately. The SLFVSP institutions were geographically located in the Northeast (33.3%), Midwest (26.7%) Southeast (20%), and West (20%). Most SLFVSPs operated in a primary care setting associated with an SRFC (53.3%). The median reported duration since beginning operations was 6 years with an interquartile range of 17 years and a range of 6 months to 51 years.

### Quantitative results: Logistics and Operations Data

Table 1 depicts summary characteristics describing the seasonal and weekly timing of services offered, documentation strategies implemented, location(s) of screenings, funding sources, and appointment workflow strategies. Most SLFVSPs (56%) reported consistent year-long operations, while an additional 19% conduct possible year-long operations based on leadership variability. There was not a preference favoring weekdays or weekends for operations. For documentation purposes, there was equal usage of paper (50%) versus electronic records (50%). Electronic record systems utilized included Epic, Practice Fusion, Kareo, and Athena.

**Table 1:** A summary depicting various logistical strategies, including total operation time, times when screenings are offered, documentation strategies, locations utilized, funding sources, and appointment type is provided. For “Location,” a clinic may have endorsed more than one site and the responses may exceed the total number of respondent SLFVSPs. Percentages are calculated out of the total number of SLFVSPs that responded to the particular item(s)

				%	*n*
*Response Rate*	**When offered**	Year-Long	Yes	56%	9
			No	25%	4
			Maybe	19%	3
16/16		Time of Week	Weekday only	50%	8
(100%)			Weekend only	44%	7
			Both	6%	1
*Response Rate*	**Documentation Strategy**	Paper		50%	8
		Electronic Health Record		50%	8
16/16 (100%)		Epic		25%	2
		PracticeFusion		25%	2
		Kareo		13%	1
		Athena		13%	1
		Unlisted		25%	2
*Response Rate*	**Location**	Federally Qualified Health Center		50%	8
		Religious Center		38%	6
		Street Outreach		13%	2
		Health Fair		6%	1
16/16		Homeless Shelter		6%	1
(100%)		Community Organizations		6%	1
		Library		6%	1
		Medical School		6%	1
*Response Rate*	**Funding**	Internal (i.e. Department)		33%	3
		External (i.e. Foundation Grants)		33%	3
		Both		22%	2
9/16		In-Kind Donations		30%	3
(56.3%)		Community Fundraising		22%	2
*Response Rate*	**Appointment Type**	Scheduled		56%	9
16/16 (100%)		Walk-In		31%	5
		Both		13%	2
		Average Wait Time		22 m (15–45 m)	
		Average Total Visit Time		56 m (15–120 m)	

Vision screenings were held by SLFVSPs (*n* = 13 reporting screening location) in a wide variety of locations with the most popular locations including Federally Qualified Health Centers (50%) and religious centers (38%), with one SLFVSP reporting use of both locations. Half of the respondents reported using more than one type of space. When estimating the average number of rooms used during a screening event, 10 SLFVSPs reported access to at least two rooms; of this group, seven programs offered dilated eye exams. At the same time, six SLFVSPs offered services with only one room available; of this group, only one program offered a dilated eye exam.

Reported funding sources for SLFVSPs (*n* = 9 responses) demonstrated that many programs relied upon external grant funding or internal funding from the ophthalmology department exclusively (33% each). Only 2 programs relied on a combination of internal and external funding. One clinic reported sharing resources with another student initiative. Only two programs reported fundraising in the community.

Scheduled appointments for screening were used by nine SLFVSPs, while five SLFVSPs reported greater than 80% of patients presenting on a walk-in basis. Only two clinics implemented a combined scheduled and walk-in strategy. There was not a significant difference in the wait time for either a scheduled or walk-in strategy, with a mean of 22 ± 11 min for programs using scheduled visits and 18 ± 7 min for programs screening walk-ins only (*p* = 0.64). Visit times, defined as door-to-door time, ranged from 15 min to 120 min. On average, dilated eye exam visits lasted 78 ± 36 min while non-dilated eye exam visits were shorter, at 34 ± 19 min (*p* = 0.02).

**Fig. 1:**
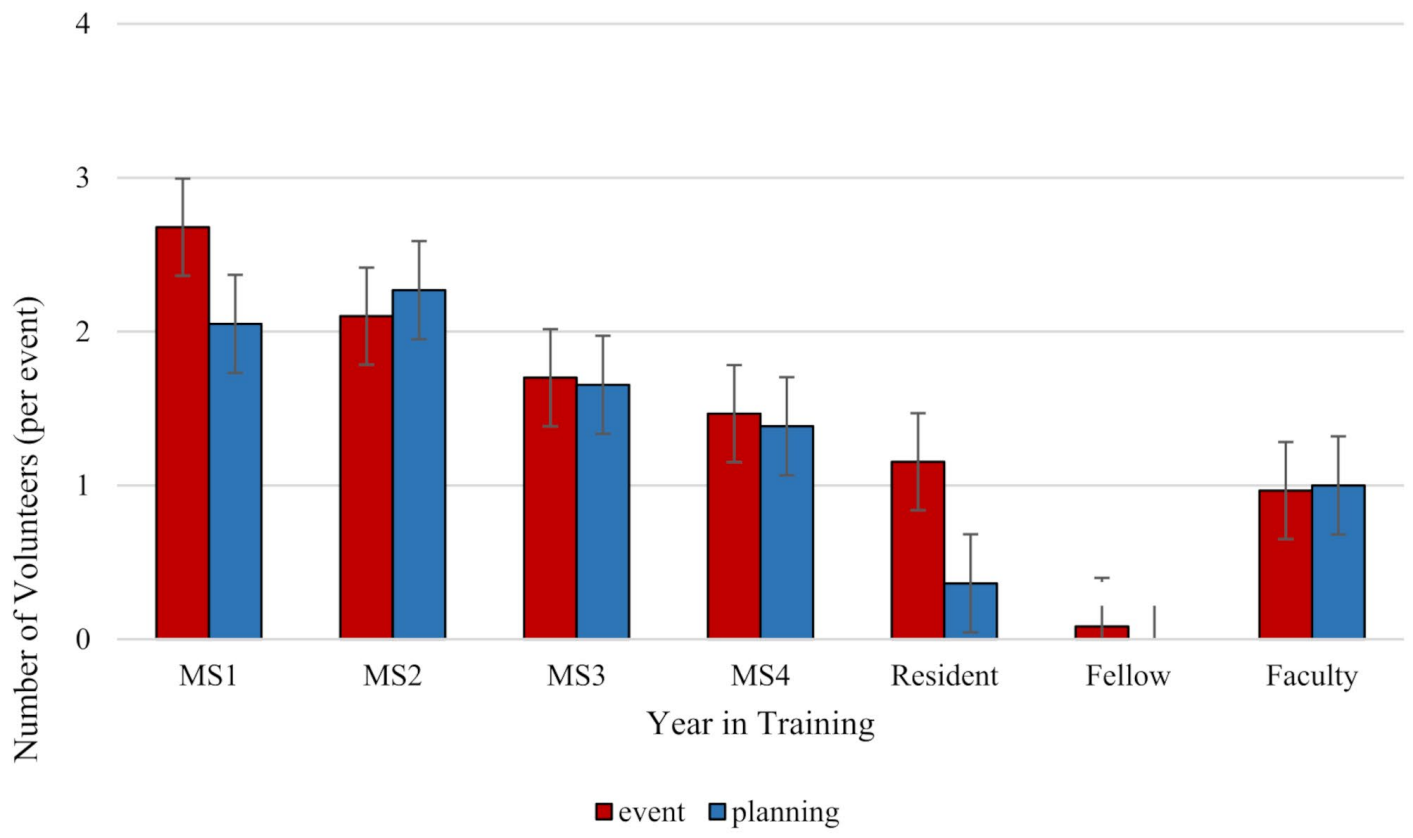
Number of volunteers per screening event stratified by training/career stage. The average number of volunteers participating in screening events (red) and event planning (blue) is shown. MS1 = first-year medical student; MS2 = second-year medical student; MS3 = third-year medical student; MS4 = fourth-year medical student. Error bars depict the standard error of the mean

Participants in clinics were largely first- and second-year medical students who were involved in volunteering and planning these programs. At one program, however, fourth-year medical students were the most involved group in the planning committee. Attendance at clinics, defined as participation in the event and engaging in planning sessions, is depicted in Fig. 1. Clinical ophthalmology fellows were the least likely to be engaged in SLFVSPs. Non-student SLFVSPs screening event involvement included faculty assistance with planning for 10 programs and resident participation for 8 programs. To plan screening sessions, SLFVSPs recruited medical students (*n* = 16), faculty (*n* = 12) and residents (*n* = 8). Less than half of the programs scheduled or advertised the clinic (*n* = 7) or needed to reserve clinic space (*n* = 6). Few clinics reported participation of community volunteers (*n* = 5), social workers (*n* = 4), nurses (*n* = 2) and pre-professional or Master of Public Health students (*n* = 2). In a series of questions considering the role of the COVID-19 pandemic on SLFVSP operations, all respondents (*n* = 6) endorsed fewer sessions with five indicating fewer patients, three indicating lower availability of faculty, two demonstrating a higher no-show rate and modified hours, and one showing lower student availability.

### Qualitative responses: perceived assets and limitations

Out of the 16 SLFVSP responses included in this study, eight defined overall strengths and seven endorsed the ability to disseminate successful component(s) of their program to a wider audience. Furthermore, eleven cited intrinsic operational weaknesses, of which seven expressed a desire for troubleshooting within a consortium setting. The aggregate of these responses is depicted in Table 2, illustrating the collective assets and liabilities of the surveyed SLFVSPs. The most reported asset (*n* = 7) centered on offering scarce vision screening services within a community that did not have consistent access to care, while the most reported liability (*n* = 5) was difficulty recruiting sufficient faculty supervision.

**Table 2:** Qualitative themes with self-reported assets and liabilities for the participating SLFVSPs, with sample quotations

Theme(s)		*n* (%)	Sample comments
**Self-Reported Assets** Total Respondents *n* = 10	Offer access to scarce vision screening services	7 (70)	*“We are capturing individuals who often have never received any vision screening before”* *“[Offer] screenings for chronic diseases (DM retinopathy*,* cataracts*,* glaucoma)”*
	Offer access to vision correction (i.e. reading or prescription glasses)	4 (40)	*“Able to provide free reading glasses and provide prescription eyeglasses through partnership with local optical vendors.”* *“Cheap eye glasses (ReSpectacle)”*
	Foster positive relationships within community	3 (30)	*“Strong partnerships with community-based organization”* *“Working with a local community church using their site as clinic”*
	Medical student education	2 (20)	*“We are also exposing M1s to ophthalmology*,* because M1s are allowed to volunteer at our weekly clinics and the***[name of student organization redacted]***board positions are given to M1s during the fall of each year.”* *“[D]evelopment of ophthalmological skills for student volunteers”*
	Long-standing experience with program implementation	1 (10)	*“long track record”*
	Expertise with Artificial Intelligence technology	1 (10)	*“With the consortium*,* we were able to share our work with AI-driven Fundus photography for Diabetic Retinopathy screen which led to a great conversation among the attendings and student volunteers”*
**Self-Reported Liabilities** Total Respondents *n* = 11	Difficulty recruiting faculty and/or resident for oversight OR community ophthalmologists	5 (45%)	*“Donation of time (faculty & residents)”* *“Maintaining staffing for regular clinics”*
	Limited or obsolete equipment	3 (27%)	*“Purchasing equipment/ repairing equipment”* *“Lack of tonopens - we have one but we need to calibrate it often.”*
	Limited space	3 (27%)	*“Lack of exam rooms. Lack of areas to transport patients while they’re dilating for clinic efficiency.”* *“Limited space”*
	Difficulty with patient recruitment	1 (9%)	*“Patient recruitment”*
	Difficulty with patient follow-up for definitive care	1 (9%)	*“Follow-up retention rate”*
	Difficulty with financially supporting program	1 (9%)	***“*** *Funding”*
	Difficulty with volunteer training	1 (9%)	*We would also like to know how other programs train their volunteers*,* given that most of our volunteers have not had prior ophthalmology experience.*

### SWOT analysis

**Fig. 2:**
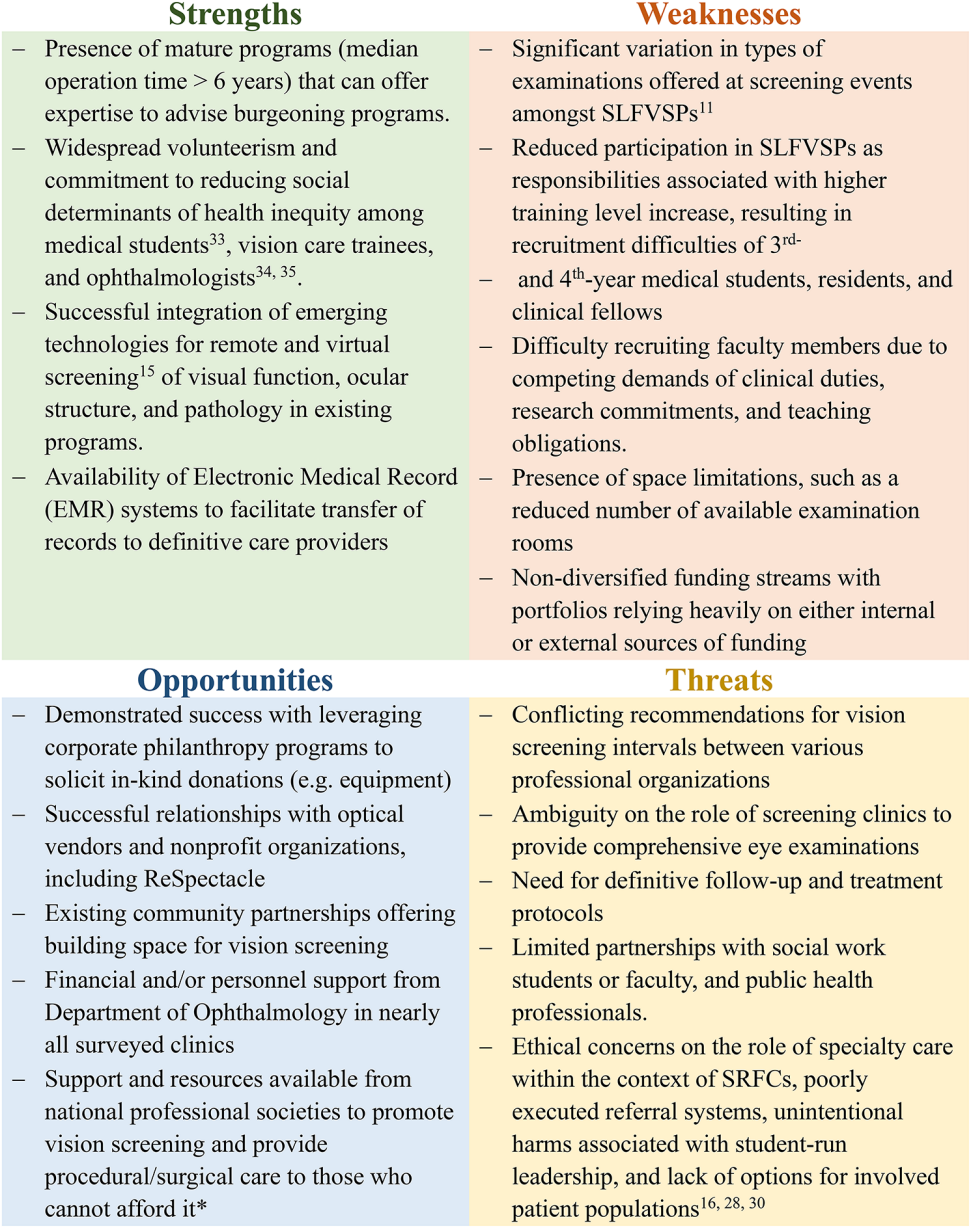
Strengths, Weaknesses, Opportunities, Threats (SWOT) analysis of establishing a nationwide consortium of student led free vision programs to foster collaboration, establish standardized protocols, and enhance equity among programs. *Lions Club, Mission Cataract USA, EyeCare America Senior, Glaucoma, and Retina Referral Programs (American Academy of Ophthalmology), AGS Cares (American Glaucoma Society), Operation Sight (American Society of Cataract and Refractive Surgery)

A SWOT analysis evaluating a potential role for a collaborative partnership, like the CSLEC, to help individual programs deliver optimal care is depicted in Fig. 2. The analysis suggests that while SLFVSP are student-led, these organizations exist within an ecosystem of partnerships with community organizations and for-profit entities and are therefore invested in by several primary stakeholders, including ophthalmology departments (whether through contributing financial support or through endorsing faculty and trainee participation). The SWOT analysis highlights traits that confer benefit to SLFVSPs, like successful community partnerships, as well as providing insights on possible areas towards which individual SLFVSPs may consider investing, such as volunteer training and sustainability.

## Discussion

### Consensus Building

The surveyed SLFVSPs reiterated strong relationships with community organizations, like religious centers, with opportunities to host vision screening services in locations that are accessible to target populations. Even though a few SLFVSPs self-identified the presence of their services as one of the only points of access to vision care within the geographic area, there have been concerns raised in the literature about whether student-run free clinics, more generally, are truly located in areas of need [16], underscoring the importance of contextualizing trends on screening failure rates or eyecare utilization with geoinformatics data [17, 18] including measures like the Area Deprivation Index (ADI) [19].

Apart from considering the location of the screening event, optimizing space may also be an important consideration. One SLFVSP specifically cited space constraints as a limitation in offering dilated eye exams efficiently, a finding that was mirrored throughout this surveyed cohort, where there was an appreciable lack of dilated eye exams offered by SLFVSPs working with < 2 rooms. This finding is not surprising; a retrospective study considering pediatric eye examinations [17] reported that larger childcare facility sizes were more optimal for vision screening and is an important consideration for planning. While only one program specifically identified a *“long track record”* as a strength, the median age of surveyed SLFVSPs was 6 years (6 months − 51 years), signifying the presence of mature programs with the potential to mentor emerging programs in optimizing screening workflows within existing spaces or seeking more conducive locations.

Even though some screening locations are integrated within the community, definitive care may not be accessible from that location; transportation has been a consistently cited barrier for receiving follow-up care after vision screening initiatives, both previously within this cohort [11] and nationally [20]. In addition, as we reported elsewhere [11], the attendance rate for definitive care, follow-up appointments reported by SLFVSPs with direct scheduling appears to result in to higher attendance rates than from those that only process referrals. Documenting the specifics of follow-up protocols and associated follow-up rates across SLFVSPs [20] is a warranted future area of study.

In the context of both free clinics and student-run clinics, it has been demonstrated that the expertise of social work is underutilized [21] while medical student volunteers in student-run clinics lack the knowledge to refer patients to definitive forms of follow-up care [22]. It may also be helpful for SLFVSPs to determine if expanding the involvement of social work faculty or student volunteers could be beneficial. Further, it is also important to understand the extent to which SLFVSPs interface with the resources offered by national professional societies (such as like American Glaucoma Society (AGS) Cares) to help connect patients to definitive care.

Within this cohort, there was self-reported heterogeneity [11] in both overall goals and modalities of screening services offered. To be truly comprehensive, SLFVSPS should offer refraction services with options for free prescription glasses, anterior segment exams, and fundus exams, rather than only evaluating measuring certain ophthalmic parameters. Understanding multiple data points can offer greater clarity; as a case in point, tonometry alone has a low sensitivity for glaucoma screening without additional information derived from optic disc assessment and perimetry [23, 24]. Moreover, minimizing methodological variability among SLFVSPs will result in more robust quality assessments in the future. For instance, if a consensus finds that visual fields should be offered (at least for a subset of patients found to be at risk for relevant diseases like glaucoma), considering the type (Humphrey versus Octopus versus FDT) is also necessary. Of course, this consideration will need to balance the impact of these testing modalities with cost and feasibility.

Though SLFVSPs intend to increase access to comprehensive preventative vision care, exclusively screening for a specific disease pathology may unintentionally become a barrier to receiving comprehensive care. If an individual believes participating in a diabetic retinopathy screening clinic is a full eye examination, this misinterpretation may inadvertently result in care delays, if the patient defers other opportunities. Moreover, disenfranchised populations, who already underutilize vision care services, will strongly benefit from a complete eye exam, rather than patchworks that focus on certain disease pathologies. Participants of focused vision screening programs should therefore be counseled on the importance of a comprehensive eye exam and be redirected or referred, as appropriate.

### Organizational stewardship

Despite the self-report of financial limitations, as described in Table 2 as a need for explicit funding (*n* = 1) or equipment (*n* = 3), the financial portfolios maintained by SLFVSPs were typically limited to internal (*n* = 3) or external (*n* = 3) sources of funding, rather than combining both sources (*n* = 2). Further, a small number of SLFVSPs engaged in community fundraising (*n* = 2) or solicited in-kind donations (*n* = 3), respectively. It should be noted that needed equipment sought by one program, an Optical Coherence Tomography (OCT) device, was obtained by another as an in-kind donation, highlighting the opportunity to collaborate with industry or private practice partners to obtain equipment as a donation, rather than as a purchase. To help better estimate funding allocation needs, one general medicine free clinic reported an itemization strategy to prioritize funding by ranking different categories of operational expenses [25] which may prove helpful for SLFVSPs, which especially depend upon reliable equipment.

Despite apparent financial limitations, four SLFVSPs endorsed support for corrective vision programs, including a *“partnership with local optical vendors*” or a “*grant to pay for all patient’s glasses*,” which highlights a benefit of sharing the costs of service delivery with other entities. In addition, half of the surveyed programs utilized an EMR system, which may be otherwise cost-prohibitive without robust financial support. With more widespread adoption of EMR [26], SLFVSPs may have opportunities for population health analyses within their individual context, and offer data to support larger, collaborative projects, especially in consideration of geographic analyses [17]. While large population datasets exist, understanding social risks from the perspectives of community data in this unique group of underserved patients can help inform local policy changes and interventions, that may not otherwise be realized [27]. Most importantly, though, the use of EMRs can provide continuity when the patient pursues definitive care.

Once protocols for standardization are formalized, organizational stewardship can occur, with an emphasis on building the financial capabilities of individual SLFVSPs. With a defined equipment list and tasks, SLFVSPs can leverage connections with for-profit entities to obtain in-kind donations or solicit glasses from non-profit organizations. Further, through creating targeted cost estimations, SLFVSPs can augment internal and external funding sources.

Though the initial model of the CSLEC has included two medical student co-directors from one institution focusing on leading knowledge-sharing discussions and developing this project with existing student leaders of member SLFVSPs, the organization will benefit from the inclusion of all interested medical students, even those who are not in formal leadership role within an institution’s SLFVSPs. Over the next 5 years, developing a standardized structure for service delivery, patient follow-up and volunteer training will facilitate improvements in financial planning and outcomes assessments, though space will continue to exist for the regional and institutional differences that may exist as far as the constitution of an event organizing team or the types of grants available, for example.

### Staff Training

One SLFVSP expressed interest in identifying how other programs engage in volunteer training, “*given that most…volunteers have not had previous ophthalmology experience.”* Within this cohort of surveyed SLFVSPs, most medical student volunteers and/or leaders were either first- or second-year students. Turnover is inherent to this model thereby necessitating greater involvement of third- and fourth-year students to facilitate sustainability, which may be achieved through implementing steering committees, as proposed by Rupert et al. [28], that outsource major decision-making and quality improvement to a body of members at higher levels of training (fourth-year medical students, residents, fellows, and faculty). Apart from training student leaders, offering dedicated didactic time focused on screening methodologies and basic ophthalmic testing interpretation to student volunteers may also be a need.

To learn about ophthalmology, medical students have been shown to increasingly invest in extra-curricular opportunities, such as volunteering in the events hosted by SLFVSPs, as time allotted to this discipline in the preclinical curriculum has decreased [29]. Because patients seeking services at free clinics may have few alternative options, several bioethical analyses have called into question the practice of trainees learning on the bodies of the socially and economically disenfranchised and the potential harms of errors that occur due to insufficient prior practice [16, 30, 31]. Implementing pre-requisite training curriculums, whether as a part of a longitudinal clerkship or extra-curricular initiatives could be an important step for volunteer quality control and may diminish the occurrence of “first-time” learning of technical skills from a vulnerable patient population.

Faculty oversight is an important factor to mitigate the inherent risks of vision screening by novice volunteers. However, similar to their student run *primary care* free clinic counterparts, this cohort of SLFVSPs also cited difficulties with faculty recruitment, which may be mediated by increasing physician burnout and the opportunity costs associated with volunteerism [32]. For example, of the five SLFVSPs that self-reported faculty recruitment as a challenge, three held operations during the weekend. As advocated for by Smith et al. [32], nearly a decade ago, greater institutional support is required to help incentivize faculty participation, which might involve counting volunteer activity towards academic promotion, as facilitating SLFVSPs is contributing a unique educational experience to trainees [33]. Near-peer training alone is not ideal for early trainees [34], underscoring the importance of faculty supervision and participation in screening events. Though trainees participating in SLFVSPs have been demonstrated to learn cultural sensitivity and gain greater understanding of inequities in healthcare access as a consequence of participation [35], faculty expertise is necessary to more intentionally impart skillsets in distress tolerance in resource-limited care settings and model interprofessional coordinated care to address the unique biopsychosocial needs of vulnerable patients [31]. 

Advocating for the integration of service-learning as a part of the medical school curriculum and/or reconsidering criteria needed for successful academic promotion could be important steps for incentivizing faculty participation and oversight while also respecting the numerous competing interests and burnout that faculty may face. While SLFVSPs largely utilize medical students as volunteers, they function as legitimate extensions of academic ophthalmology departments that prioritize increasing access to vision care in their local communities; faculty must be better supported and incentivized to support these goals. To delineate the roles of medical students, residents and faculty volunteers within a consistent framework, volunteer training materials and protocols that include assessment should also be developed.

### Role for collaboration

**Fig. 3:**
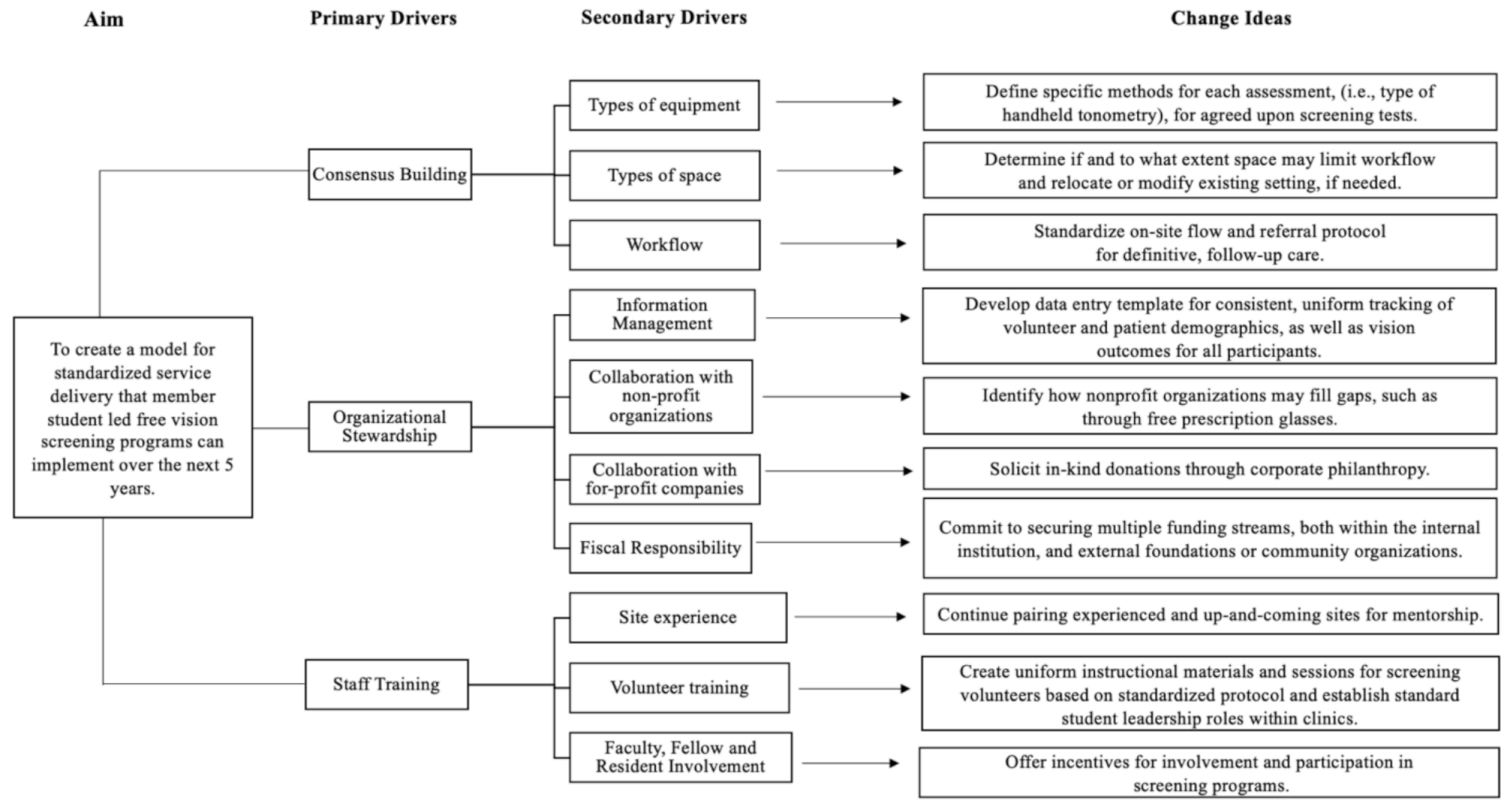
A driver diagram highlights the factors that need to be addressed to implement a standardized, delivery of screening events offered by SLFVSPs over the next 5 years. Primary drivers identified include consensus building, organizational stewardship and staff training, which will be the main areas to which attention must be directed

The CSLEC was designed to help SLFVSPs improve their ability to offer comprehensive vision screening and definitive follow-up care and to standardize operations across member institutions to allow for data-sharing necessary to evaluate clinical and public health outcomes for participating patients. A scientific approach to the appraisal of SLFVSPs was taken to first understand the capabilities and limitations of these programs, and then, to generate recommendations for the CSLEC to facilitate the focused improvement of service delivery. A driver diagram outlining the variables that must be considered to reach this goal is depicted in Fig. 3.

While the creation of an inter-institutional body to coordinate and study SLFVSPs is unique, the utilization of SWOT analyses to inform best practices for student run free clinics has precedence in the literature, with the East Harlem Health Outreach Partnership Consulting Group working to provide analyses for free clinics seeking assistance through a consultant-based model [36]. Additionally, the Crimson Care Collaborative sought to create a standardized curriculum for affiliate programs while accounting for flexibility in the realities of individual, member clinics; for example, incarcerated and immigrant patient populations necessarily have unique needs [37]. These groups both highlight potentials for collaboration to provide innovative solutions and standardization. To guide specific action steps from these *change ideas*, recommendations for both individual SLFVSPs and the CSLEC, as an organization, to improve vision care delivery are outlined in Table 3.

**Table 3:** Recommendations for the CSLEC and SLFVSPs are provided

CSLEC Recommendations	SLFVSP Recommendations
**The CSLEC should develop consensus-based guidelines to describe the scope of a comprehensive eye exam**,** design a list of acceptable testing strategies/instrumentation**,** and facilitate a template for standardizing workflow.**	If a SLFVSP is unable to offer complete eye exam or targets a specific disease pathology, i.e. diabetic retinopathy, patients should receive a combination of verbal and written counseling, as appropriate, emphasizing that they would still require a comprehensive eye exam. Specific directions and resources to obtain a comprehensive eye exam must also be provided.
	Location(s) of SLFVSP screening events should be ascertained by a geographic-based needs assessment and communication with community leaders.
	If possible, SLFVSP should investigate methods to incorporate refraction services, and/or identify partners for delivering corrective vision services at subsidized or no cost to patients.
	If possible, SLFVSP should consider the feasibility of dilated eye exams within their context, to help guide decision making.
**The CSLEC should invest in consensus-based guidelines for sending patients to definitive care**,** delineating what can be acceptably treated on-site**,** and developing a referral database of providers throughout the network.**	SLFVSP may consider implementing a mechanism of direct appointment scheduling for patients, as opposed to referrals.
	Within the framework of screening events, SLFVSP may benefit from including the input and participation of social work students and faculty to navigate barriers for patients based on their expertise.
**CSLEC should define research priorities especially to ascertain impact longitudinally considering variables like service delivery**,** baseline characteristics**,** and optimal functioning of programs.**	SLFVSP should conduct data stewardship practices that facilitate individual and collaborative prospective study designs, through investing in EMR or other similar applications.
	SLFVSP can consider collaborating with bioethics/ medical humanities departments, as well as faculty engaged with Diversity, Equity and Inclusion (DEI) to audit practices in real-time.
**CSLEC should develop consensus-based curriculums that can be adaptable to individual settings and yet attend to the quality control measures for student**,** resident/fellow**,** and faculty volunteers.**	SLFVSP can customize written materials that inform tasks of student leaders, volunteers, residents and participating faculty to offer consistency.
	To reduce sustainability problems with student leadership turnover, SLVSPs can internally develop steering committee with fourth-year medical students, residents, and faculty.
	Through working with the Medical Student Educator (MSE) within Ophthalmology Departments, SLFVSPs can consider co-curricular or extra-curricular volunteer training.
**CSLEC should work with leaders in academic ophthalmology to help determine optimal ways to integrate student-run free clinics with department goals and incentivize faculty participation with advancement.**	In working with the leadership of Ophthalmology Departments, SLFVSP can identify how faculty volunteer roles correspond to existing elements needed for academic promotion and itemize faculty contributions.
	SLFVSP can draw faculty with clinical interests in this area to volunteer and teach.

While SLFVSPs were self-aware of many of their strengths and weaknesses, aggregate analyses helped identify blind spots that may have not otherwise been identified, highlighting an advantage of conducting collective appraisals of service delivery. Individual organizations conducting SWOT analyses on their operations may be prone to cognitive errors; though self-identified assets and liabilities helped inform the foundation of the analysis in this work, studying the associated quantitative data in aggregate and in relation to previously reported information addressed the specific concerns of overconfidence and optimism bias [38]. Through participating in a collaborative trouble-shooting environment, SLFVSPs may augment their understanding of their specific circumstances and contextualize service delivery in novel manners.

Furthermore, there is also opportunity for knowledge-sharing between SLFVSPs and other types of community vision screening programs, including those focused on pediatric [39–41] or elderly [42–44] populations, though importantly, these delivery models are necessarily distinct. In addition, though other community vision screening efforts include volunteers without a healthcare background [40], SLFVSPs depend on the contributions of trainees and faculty in different health professions, requiring a different approach to volunteer training. Just as pediatric vision screening programs partner with school districts and city public health departments [41], SLFVSPs may similarly collaborate with community health stakeholders to identify key locations (including homeless shelters [45]) to host screening events. Given the challenges of associated with pursuing vision care, there are also efforts to integrate vision screening through telemedicine with primary care visits at trusted community centers [46].

At the same time, the aggregate information in this analysis was limited by the absence of contributions from optometry schools, as well as a low response rate [11]. With a small sample size, the findings of this study is not generalizable, especially as there could be a component of a non-response bias. Most of the respondents were already part of CSLEC and may have been incentivized to participate in collective quality improvement measures; the characteristics of the participants in this study may be distinct from the non-participants. Additionally, the timing of the survey during COVID-19 pandemic resulted in changes to SLFVP operations that may have made it difficult to adequately answer the questionnaire.

Factors that may have limited the response rate include survey length and breadth, unavailable data points from queried SLFVSPs, lack of tangible incentives, and the reliance on email and word-of-mouth efforts. Further, despite leveraging contact channels as outlined in the Methods section, it is unclear what percentage of SLFVSPs even received information regarding the survey; indeed, online information about some SLFVSPs were absent, outdated, or inaccurate.

## Conclusions

This study underscores the diverse operational characteristics and challenges faced by SLFVSPs across the country. The findings indicate that while these programs significantly improve access to vision care, there is a critical need for standardization in service delivery to ensure comprehensive care. A consensus should be built around the essential components of vision screenings, advocating for the inclusion of refraction services and comprehensive eye examinations to prevent misinterpretations of care. At the same time, there should be flexibility in clinic service delivery models to best fit each respective community’s needs. Furthermore, enhancing organizational stewardship through improved funding strategies and volunteer training can bolster the sustainability of SLFVSPs. By fostering collaboration and leveraging community partnerships, these programs can optimize their impact on underserved populations, ultimately advancing health equity in vision care.

## Electronic supplementary material

Below is the link to the electronic supplementary material.

Supplementary Material 1

## Data Availability

No datasets were generated or analysed during the current study.

## Abbreviatons

CSLEC: Consortium of Student Led Eye Clinics.
SLFVSP: Student Led Free Vision Screening Programs
SWOT: Strengths, Weaknesses, Opportunities, & Threats
