# Supplementary figures and images for: Recommendations for the optimization of student led free vision screening programs
Source: BMC Med Educ. 2024 Dec 18;24:1432. doi: 10.1186/s12909-024-06396-w (PMC11653908; doi:10.1186/s12909-024-06396-w)

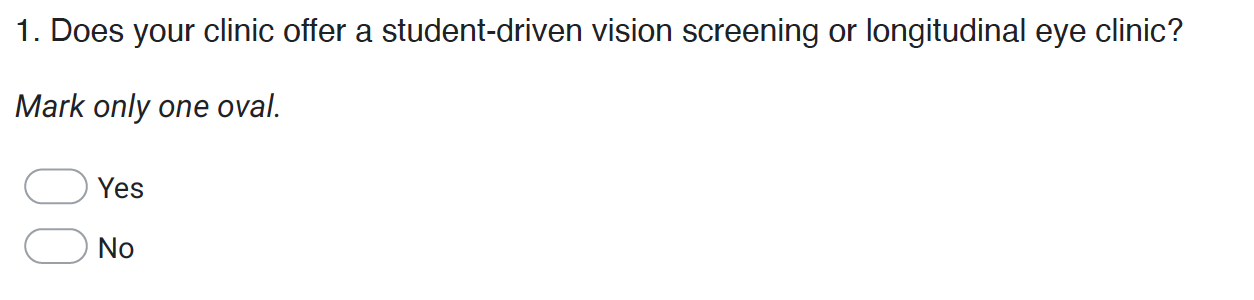


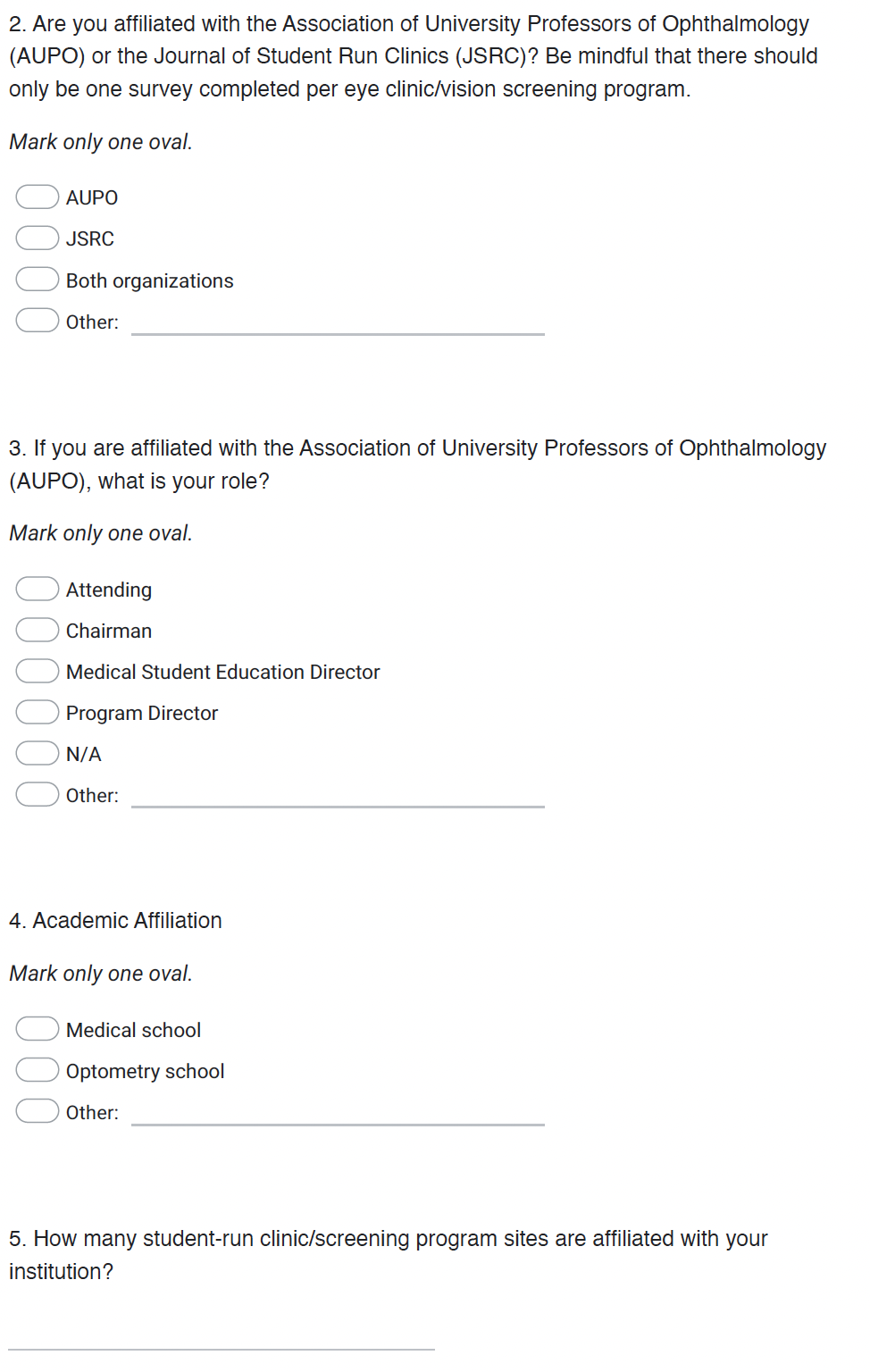


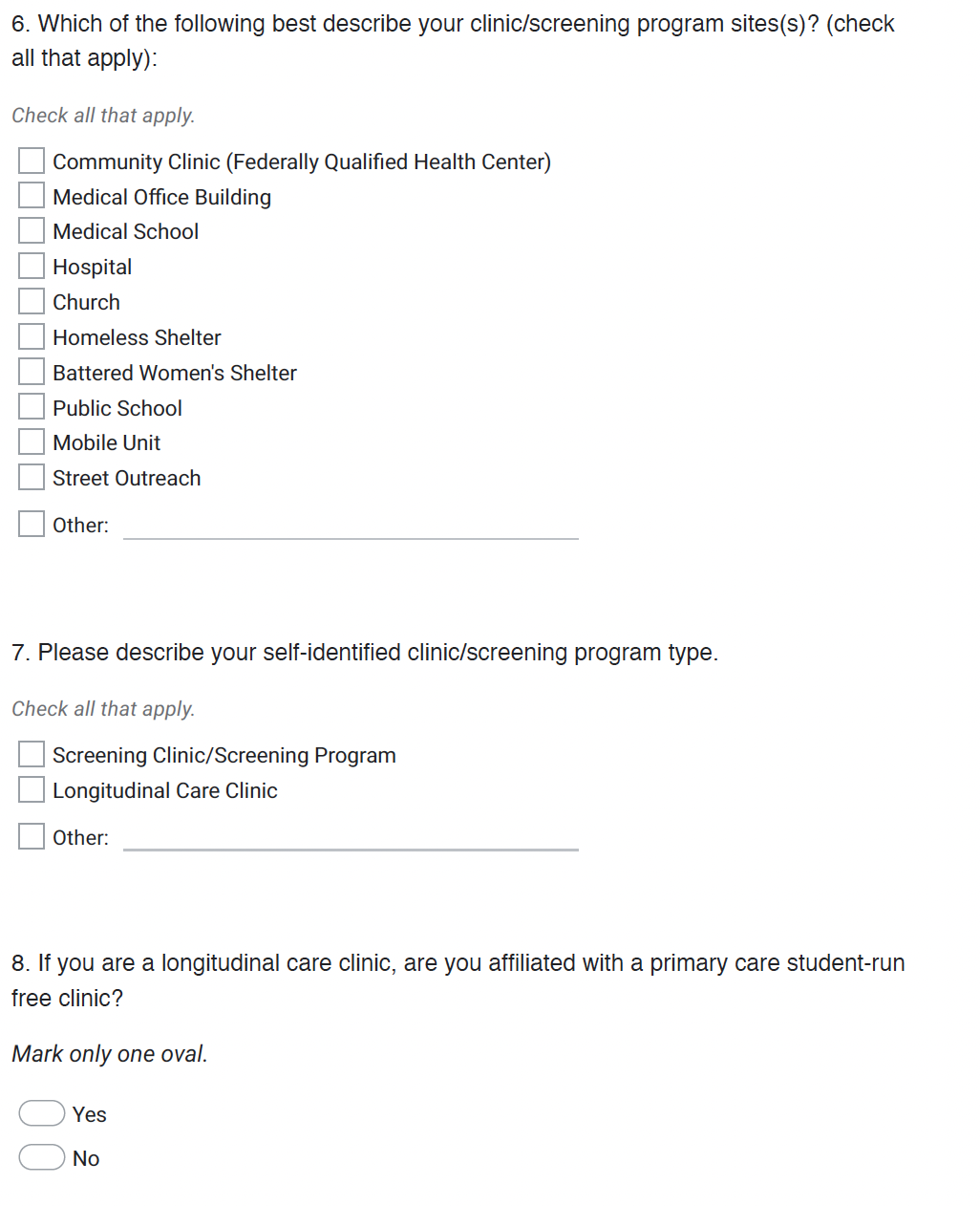


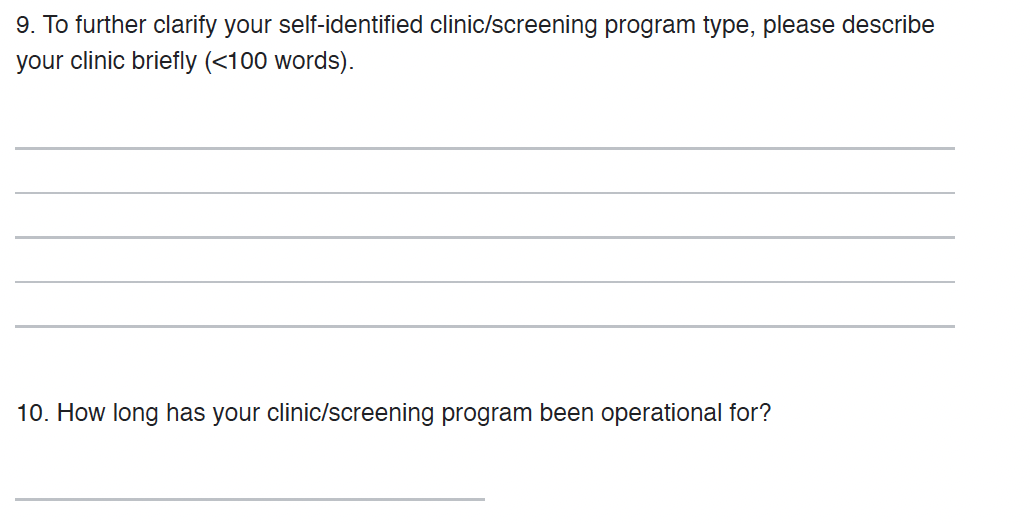


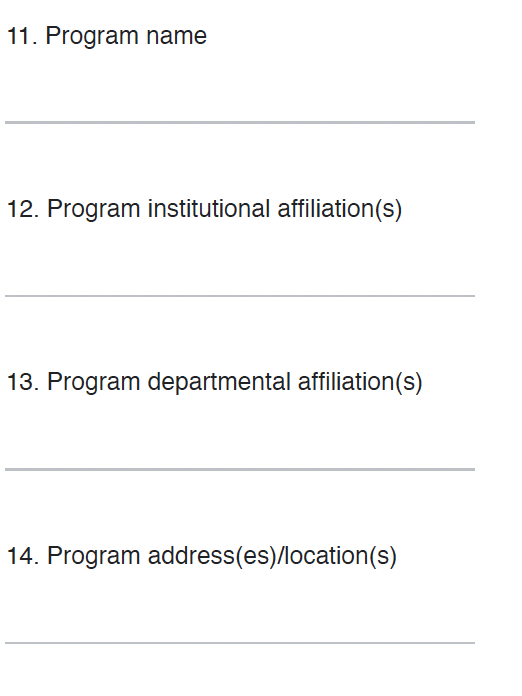

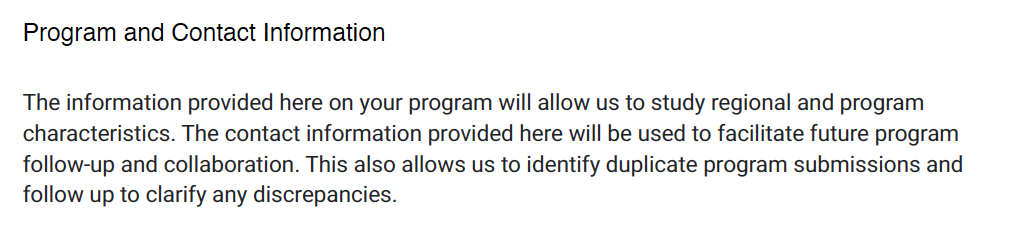


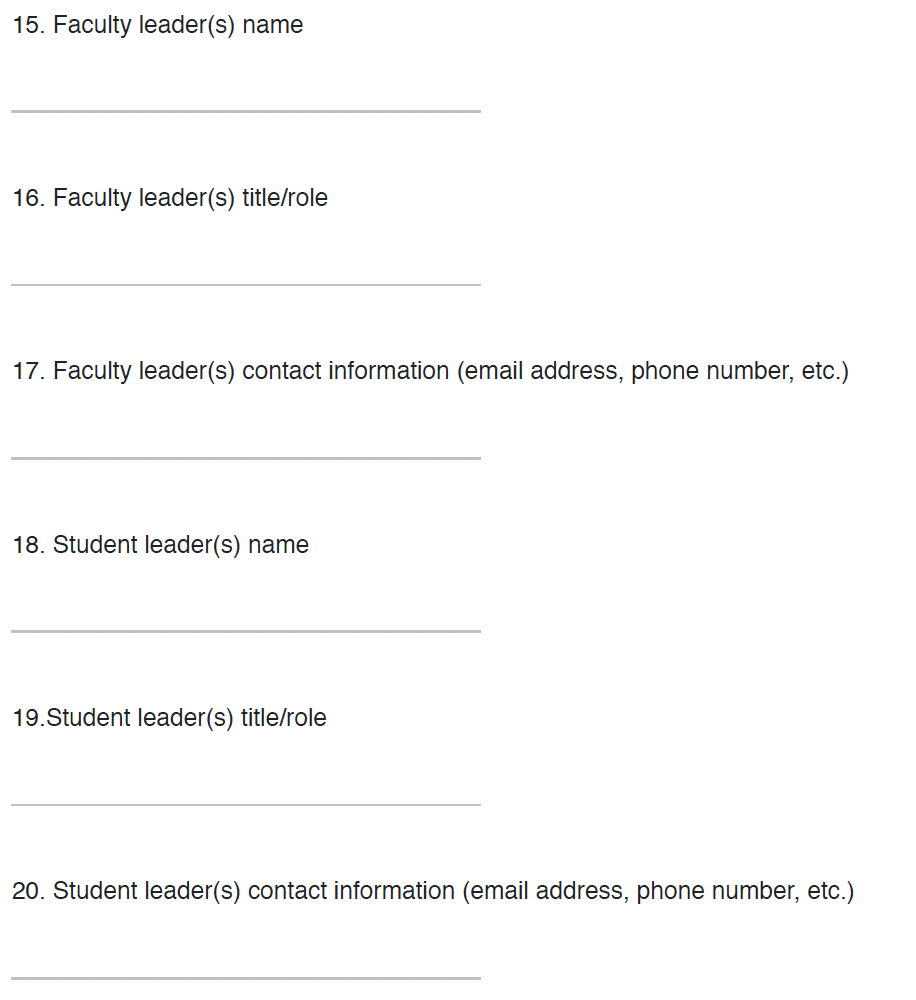


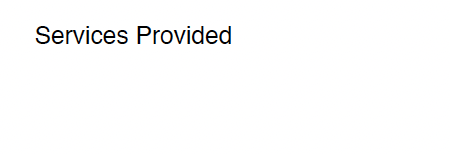


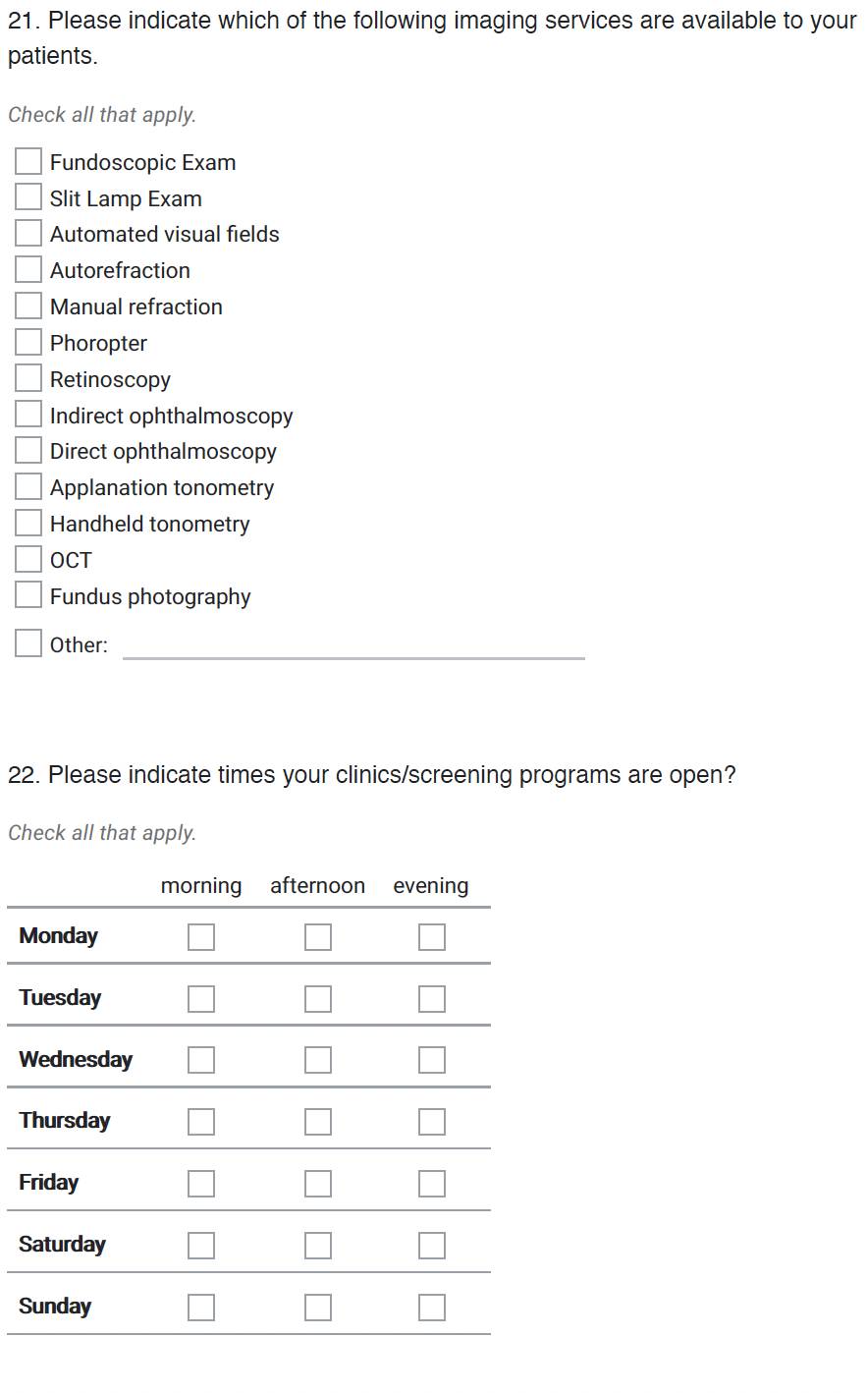


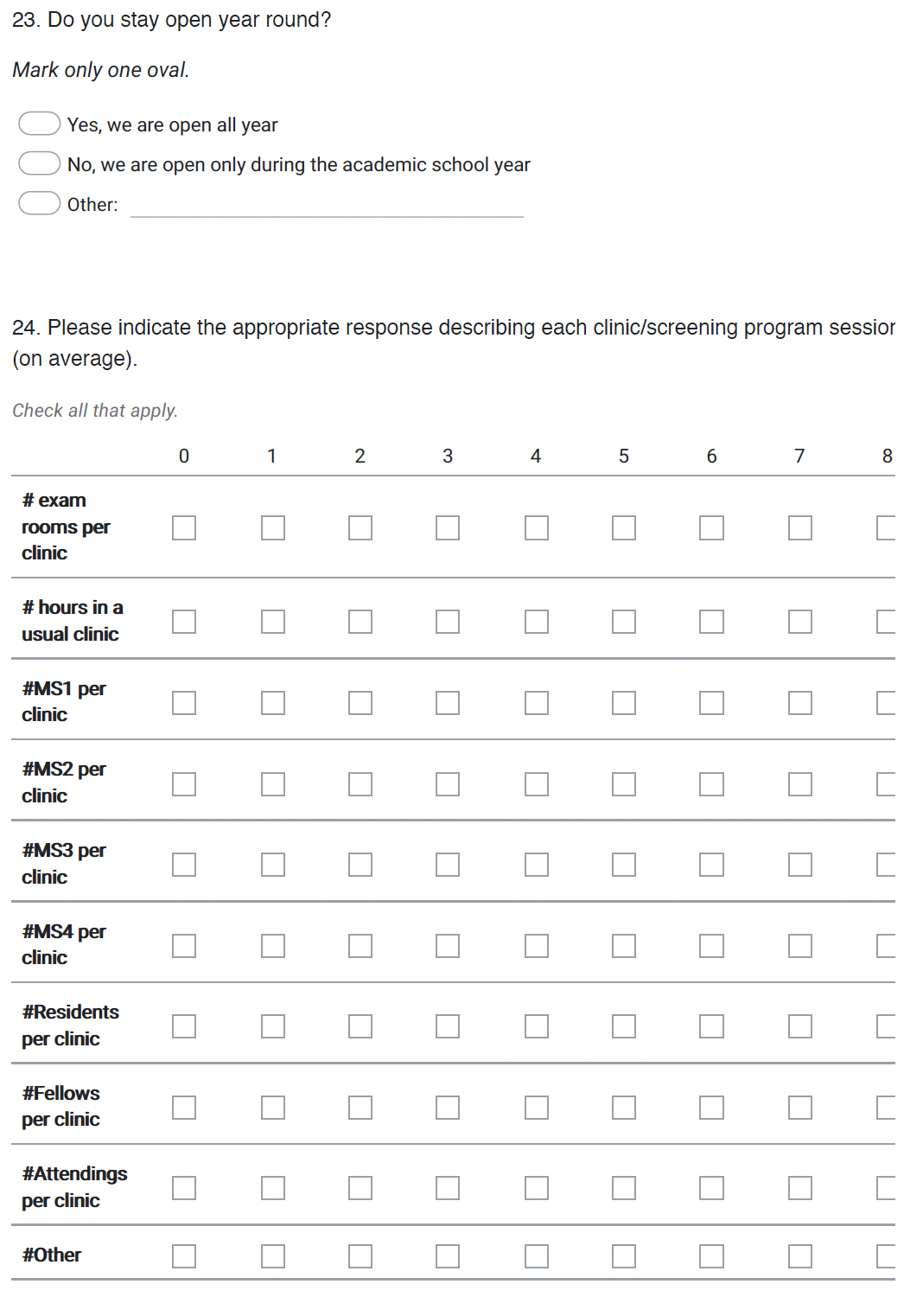


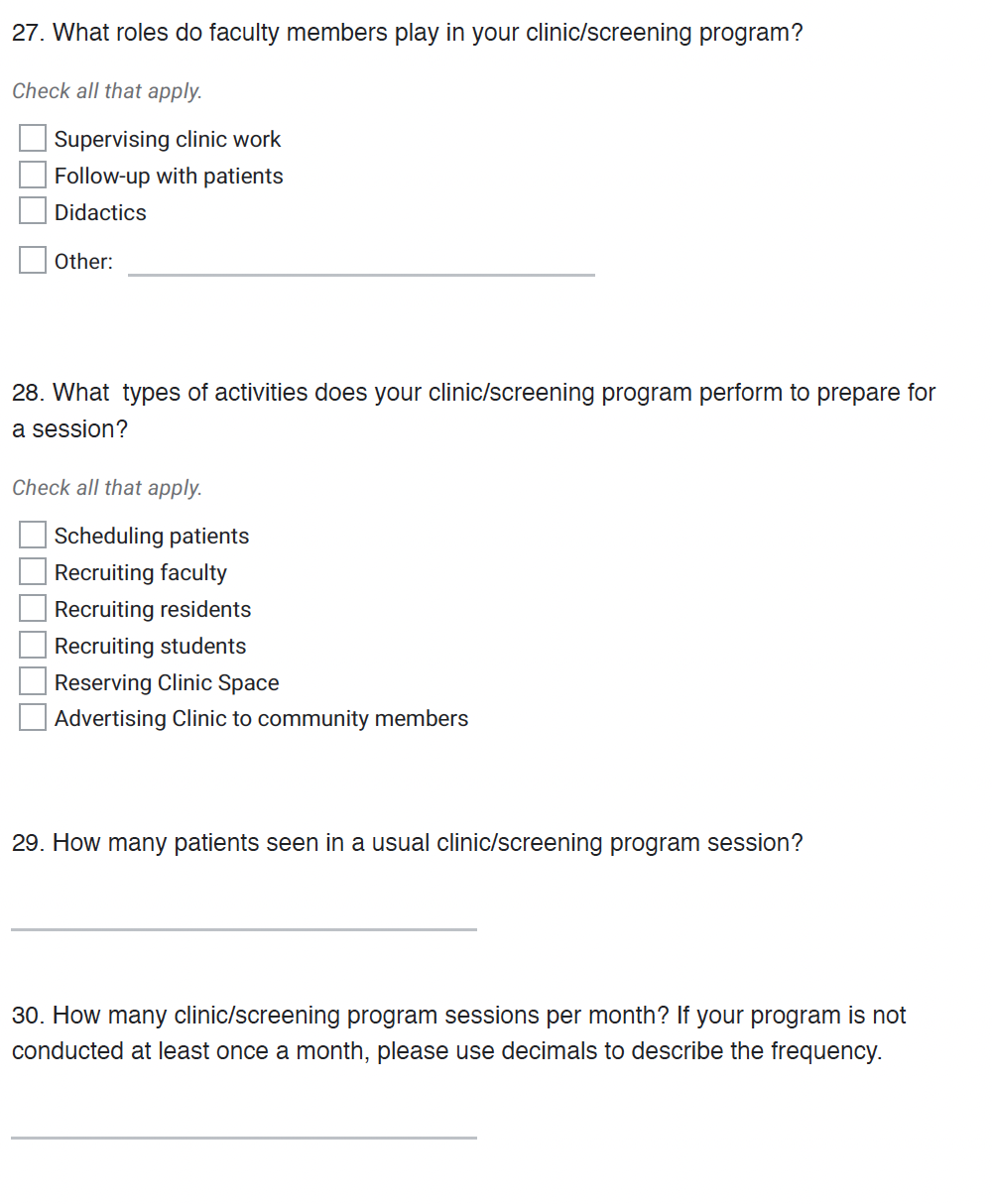

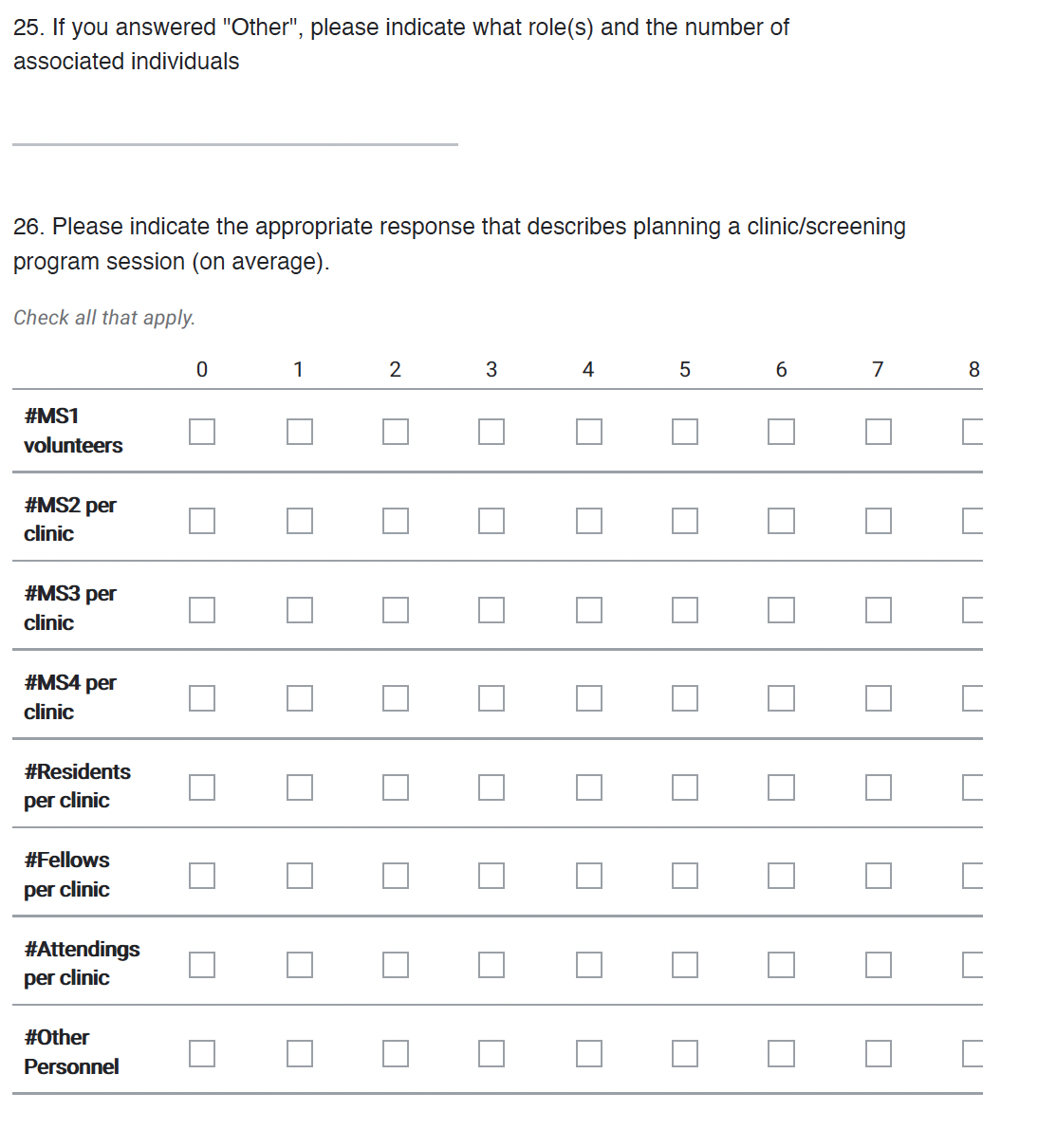


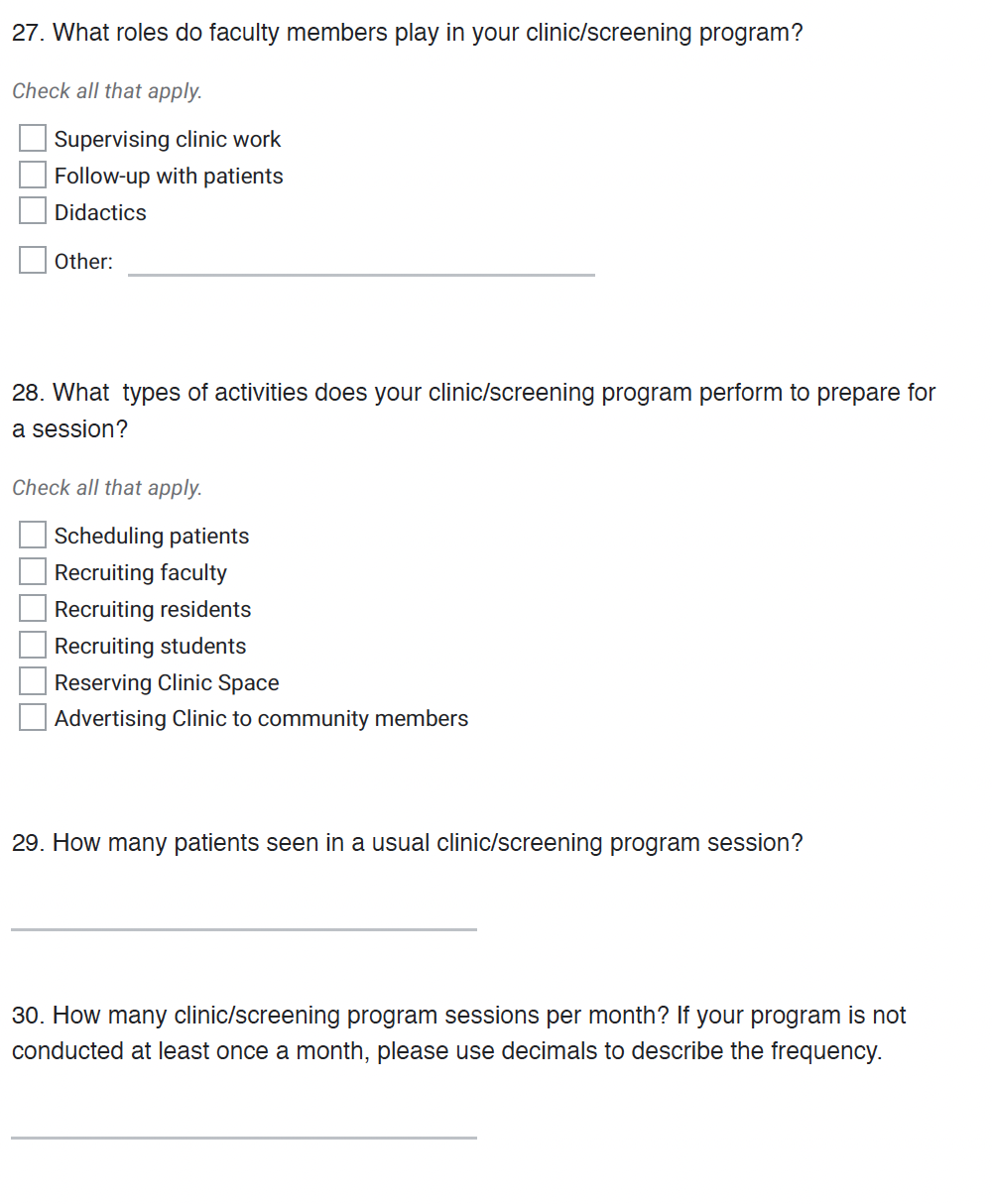


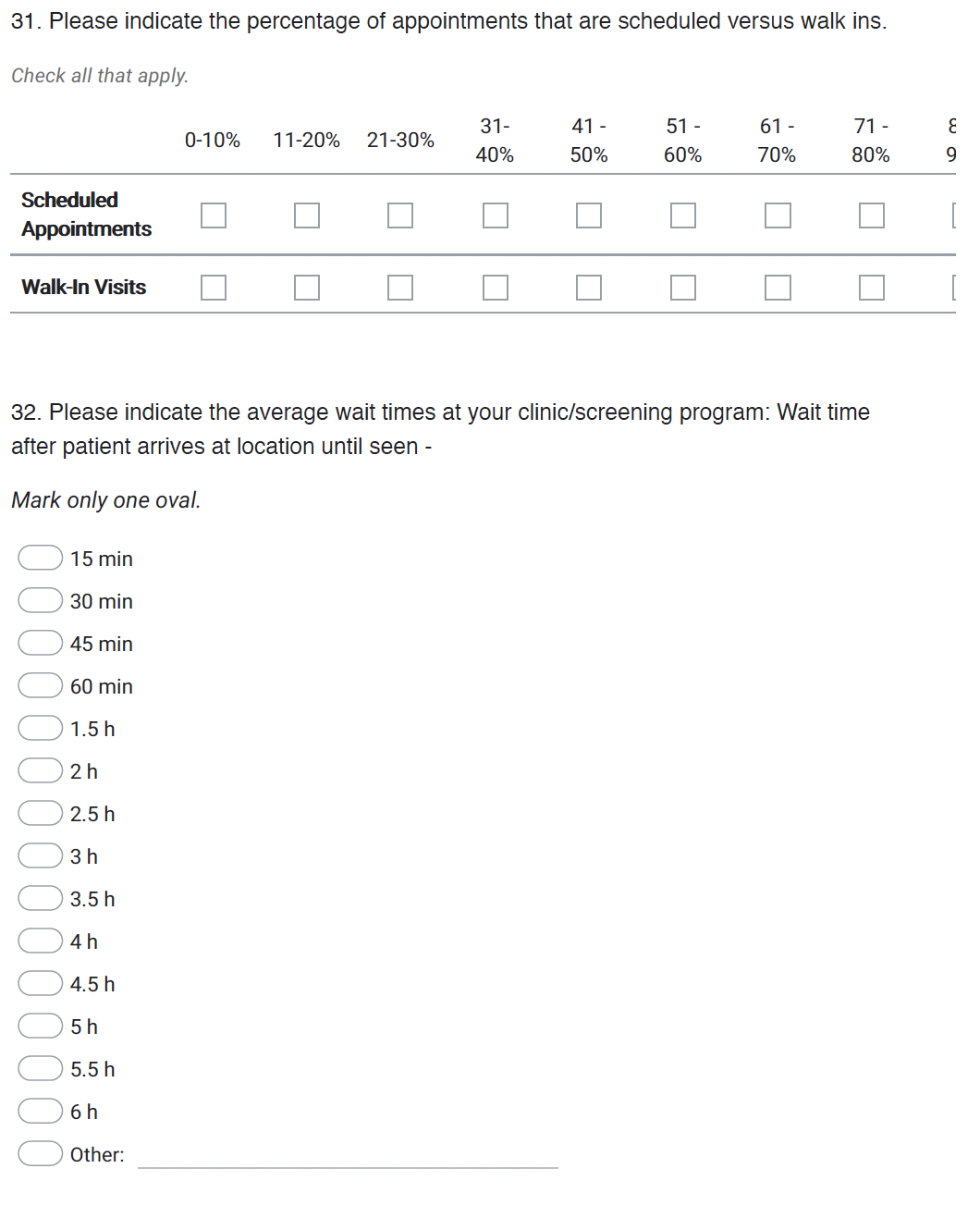


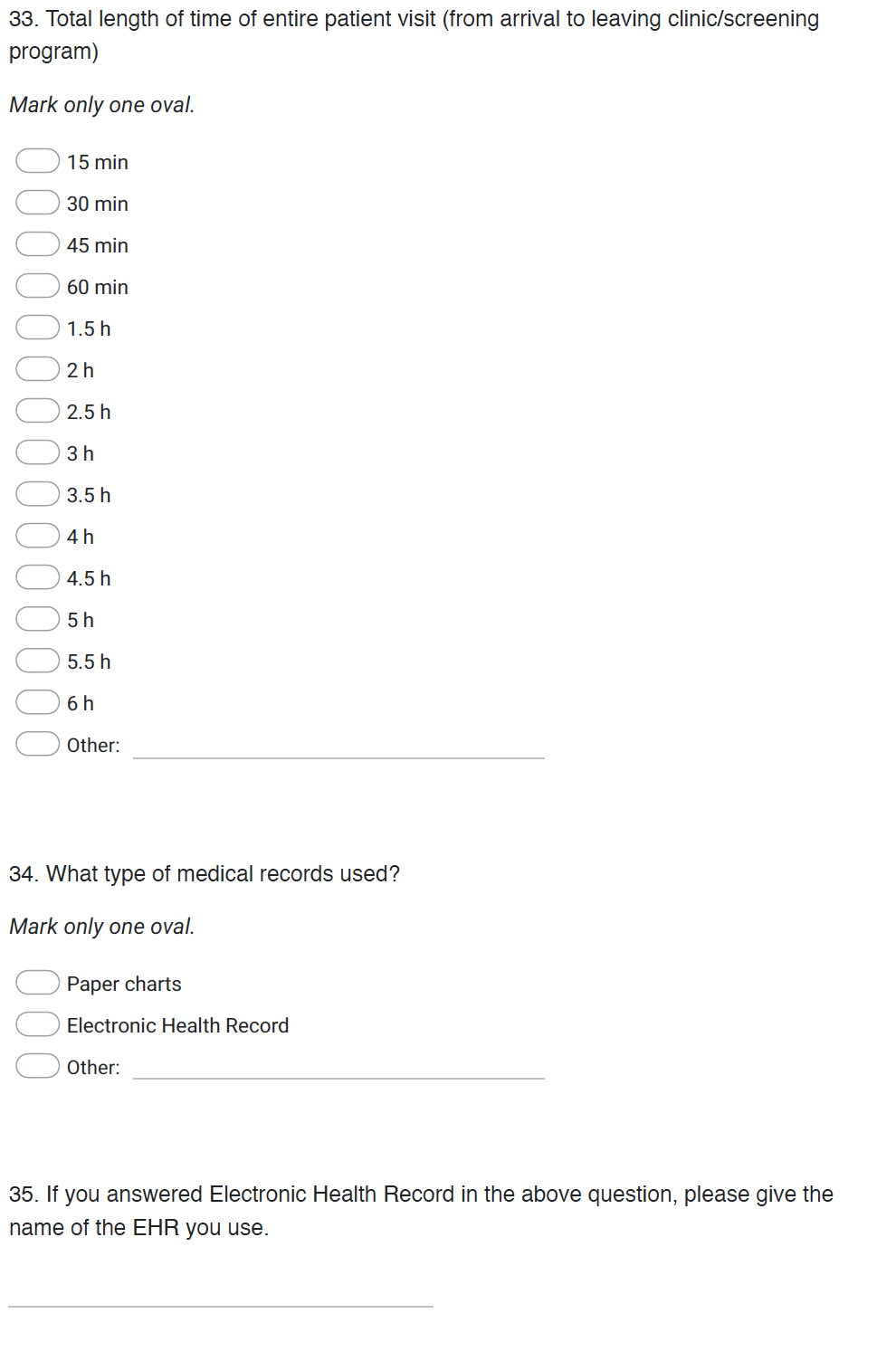


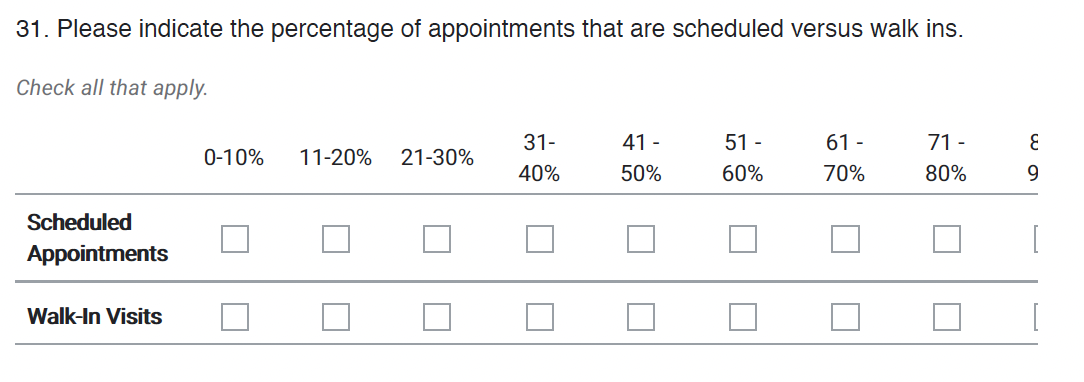

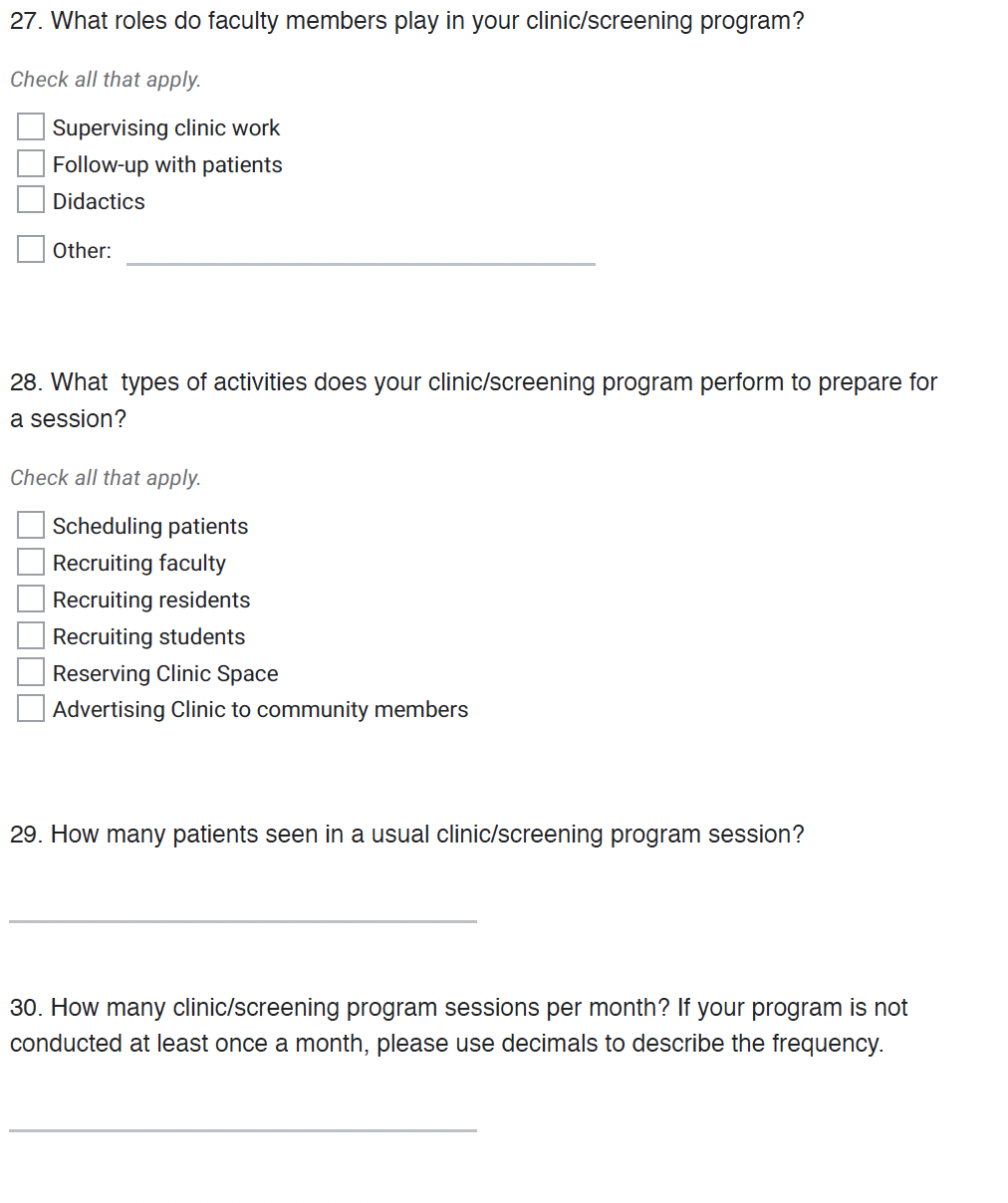

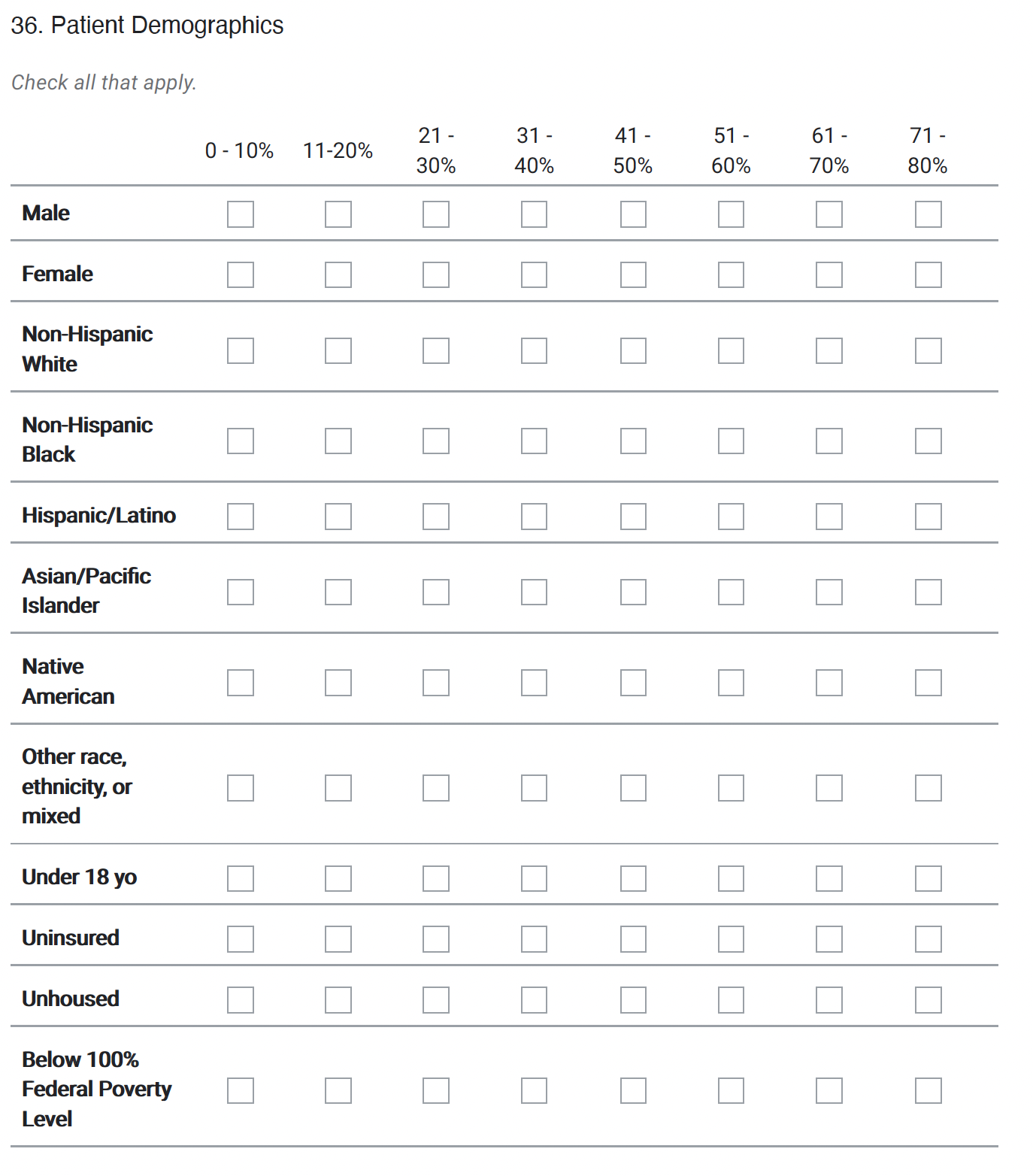


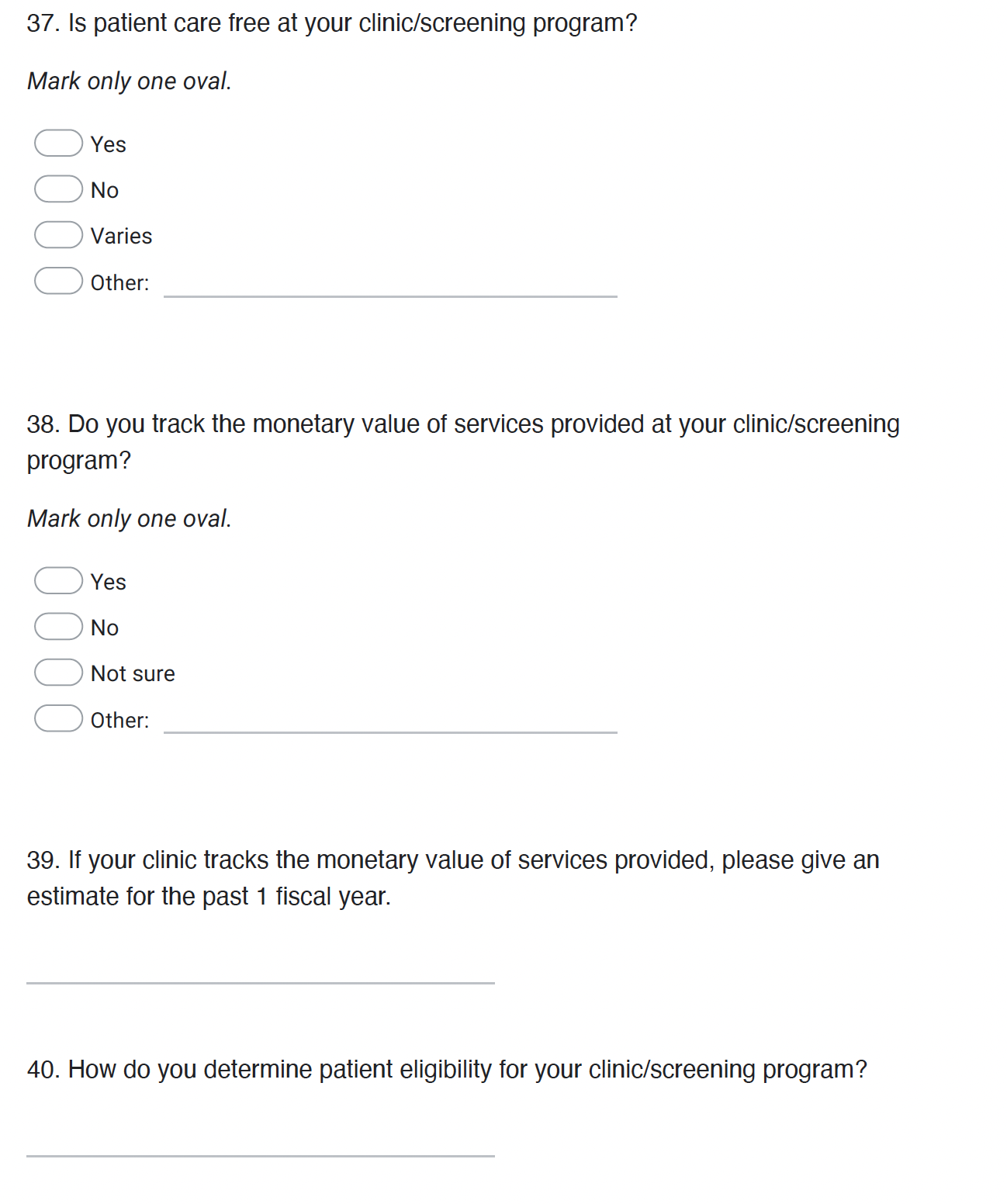


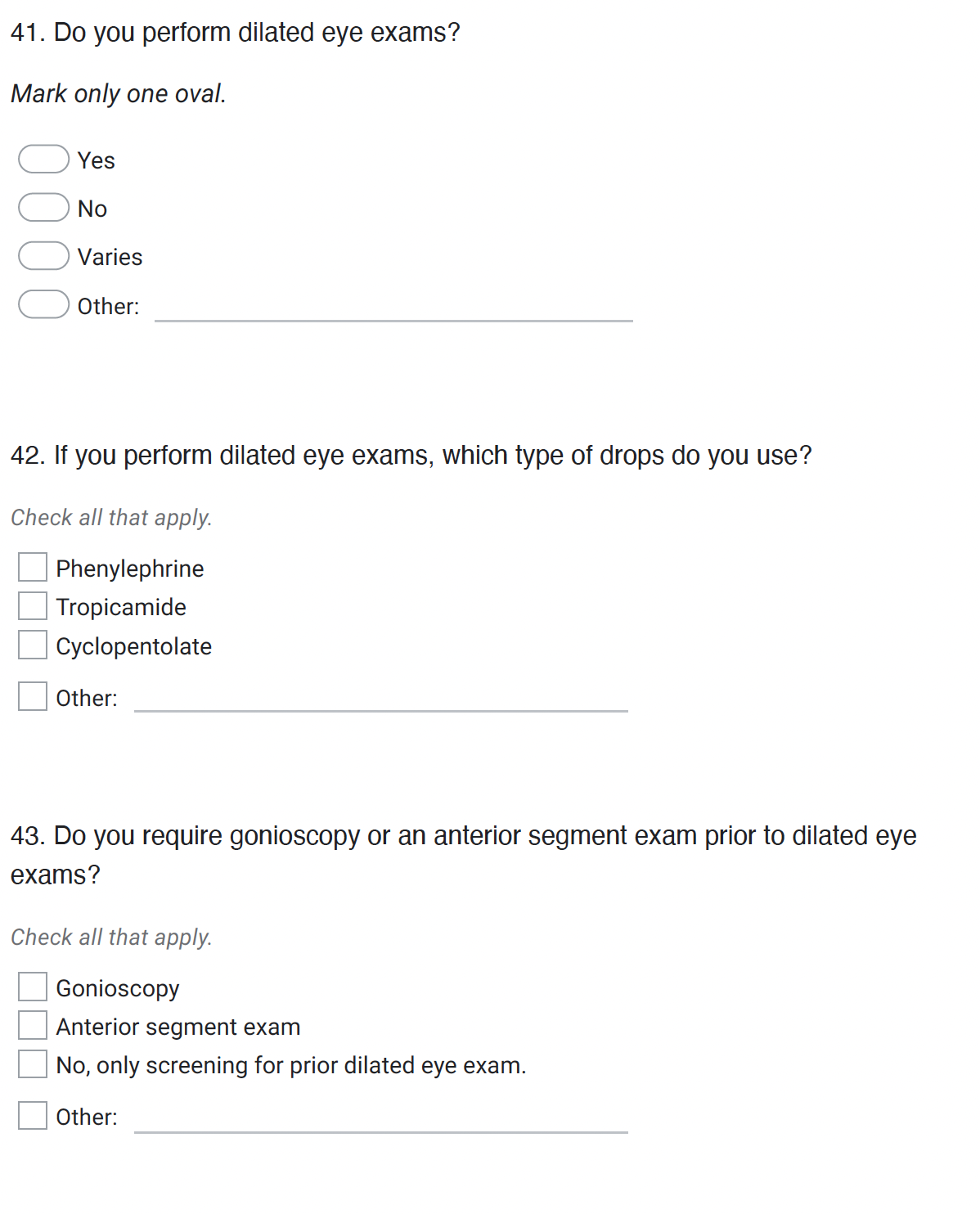


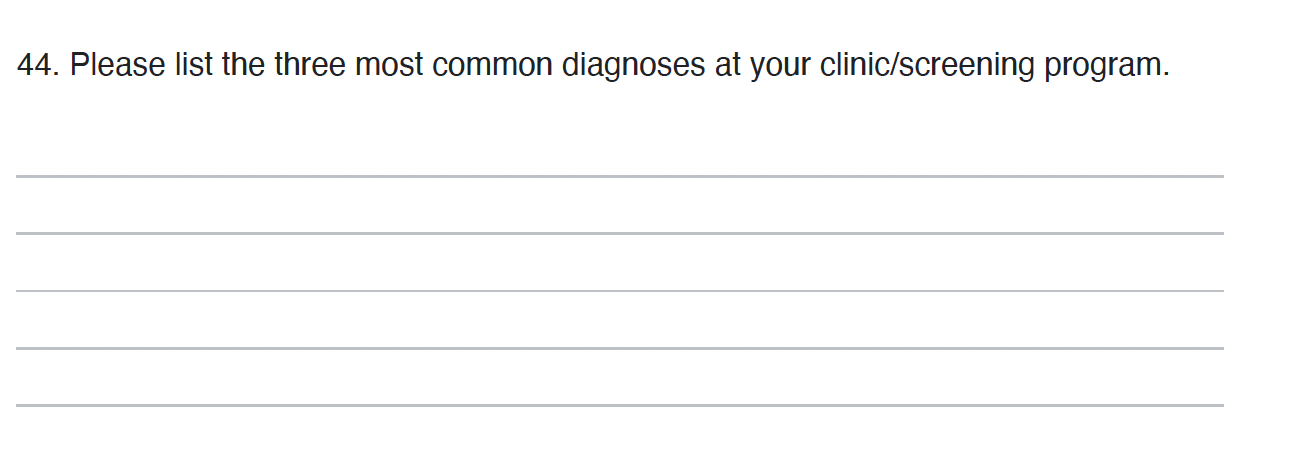


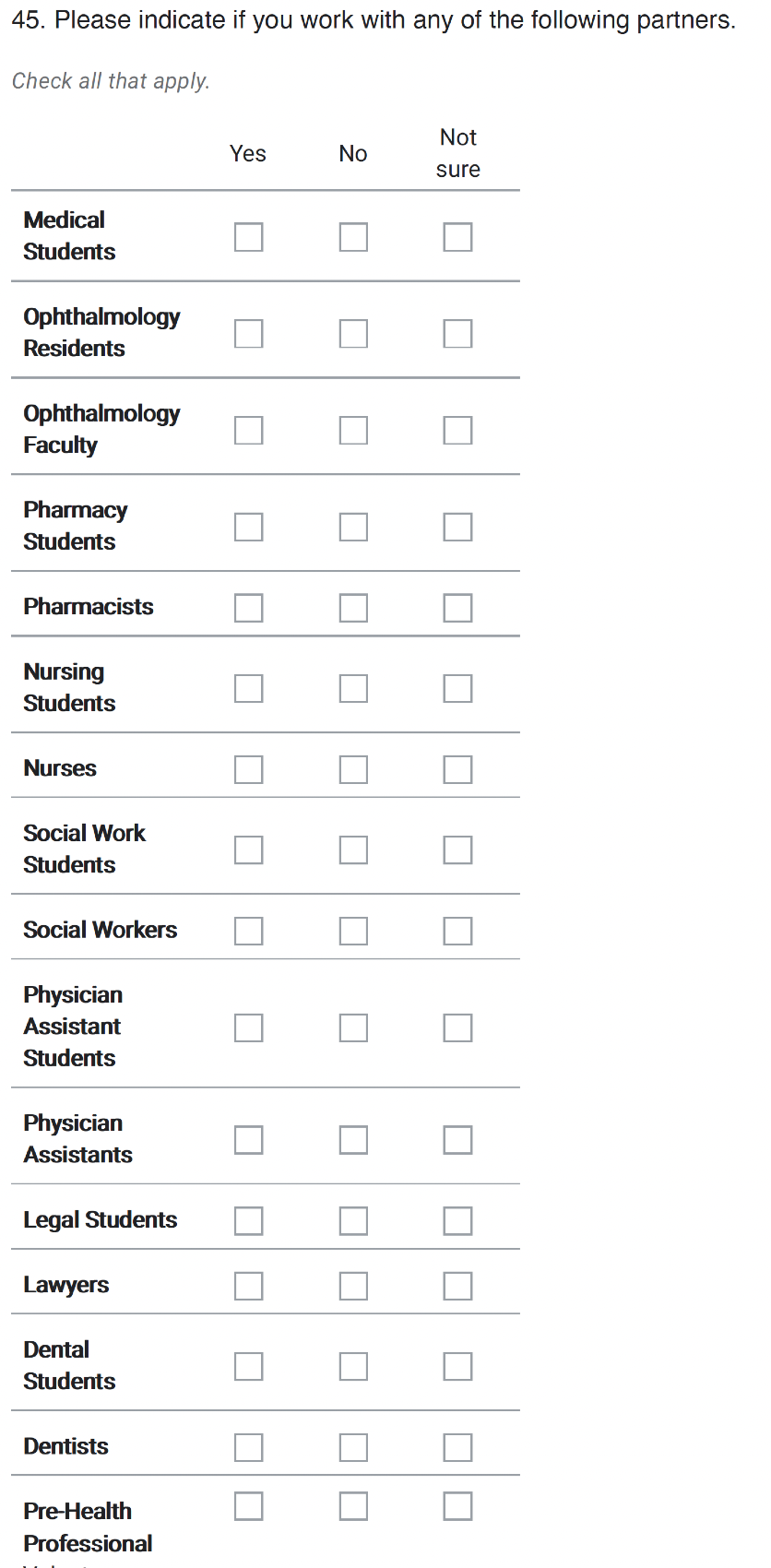


**Community Health Volunteers**


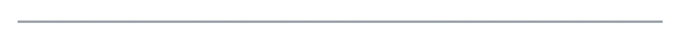

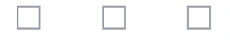

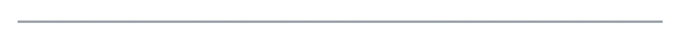

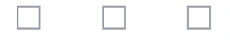


**Public Health Students (MPH)**


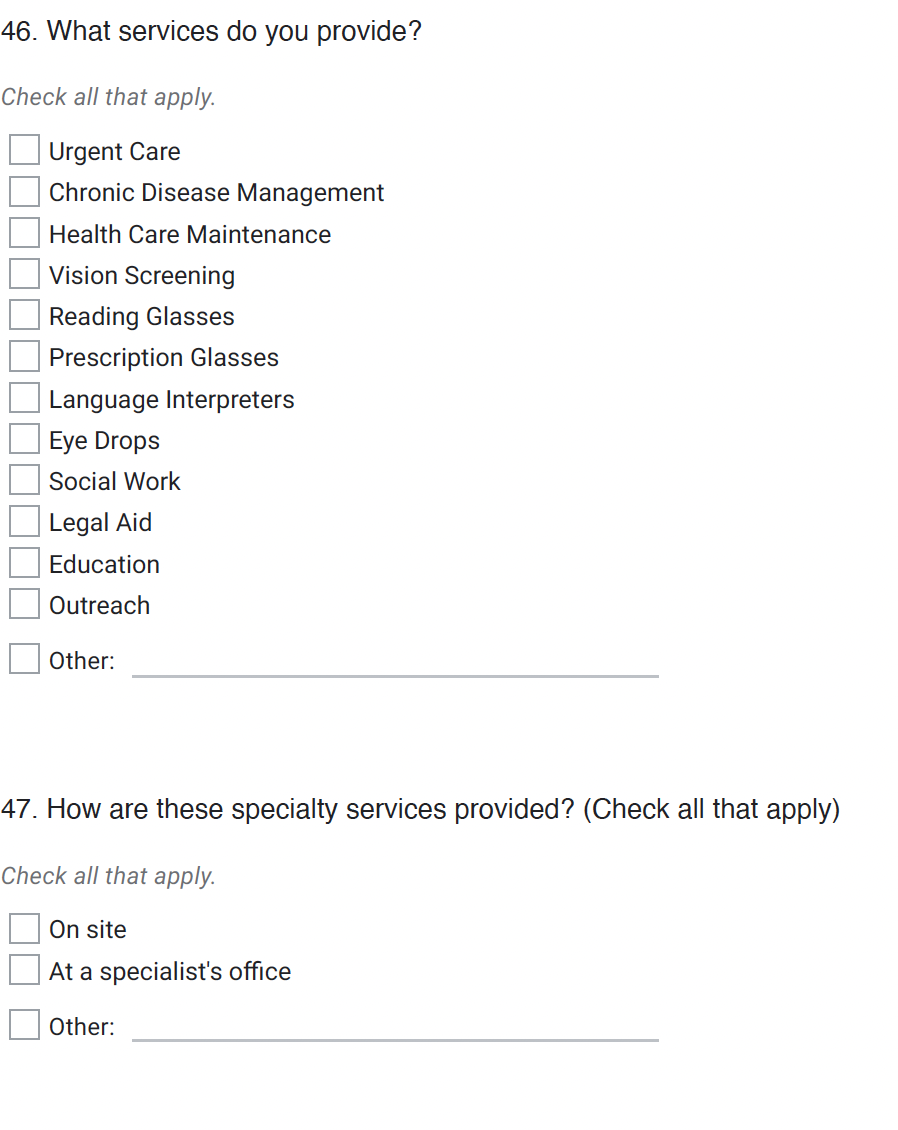


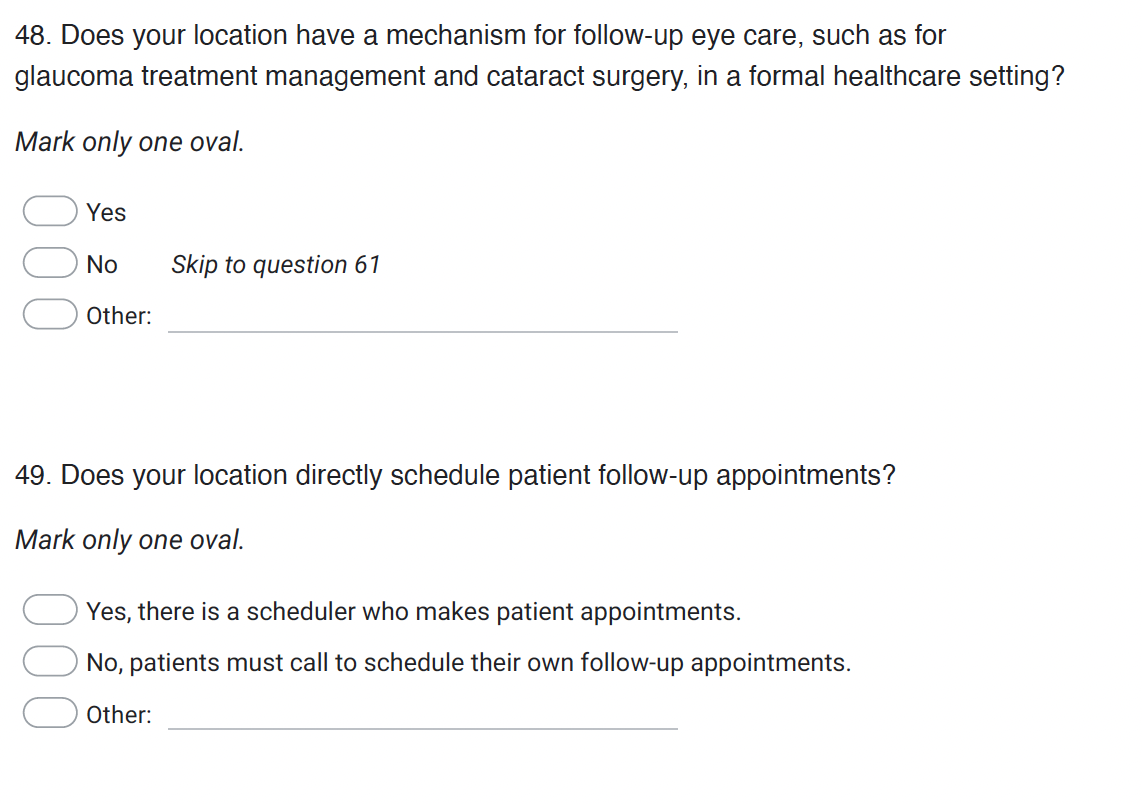


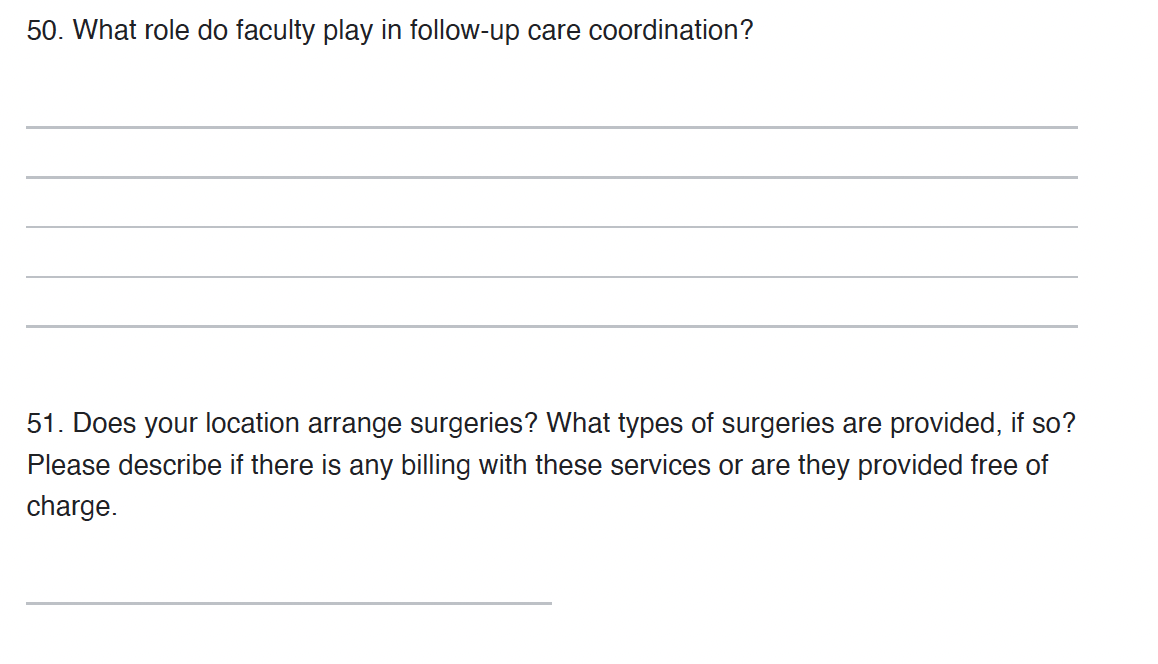


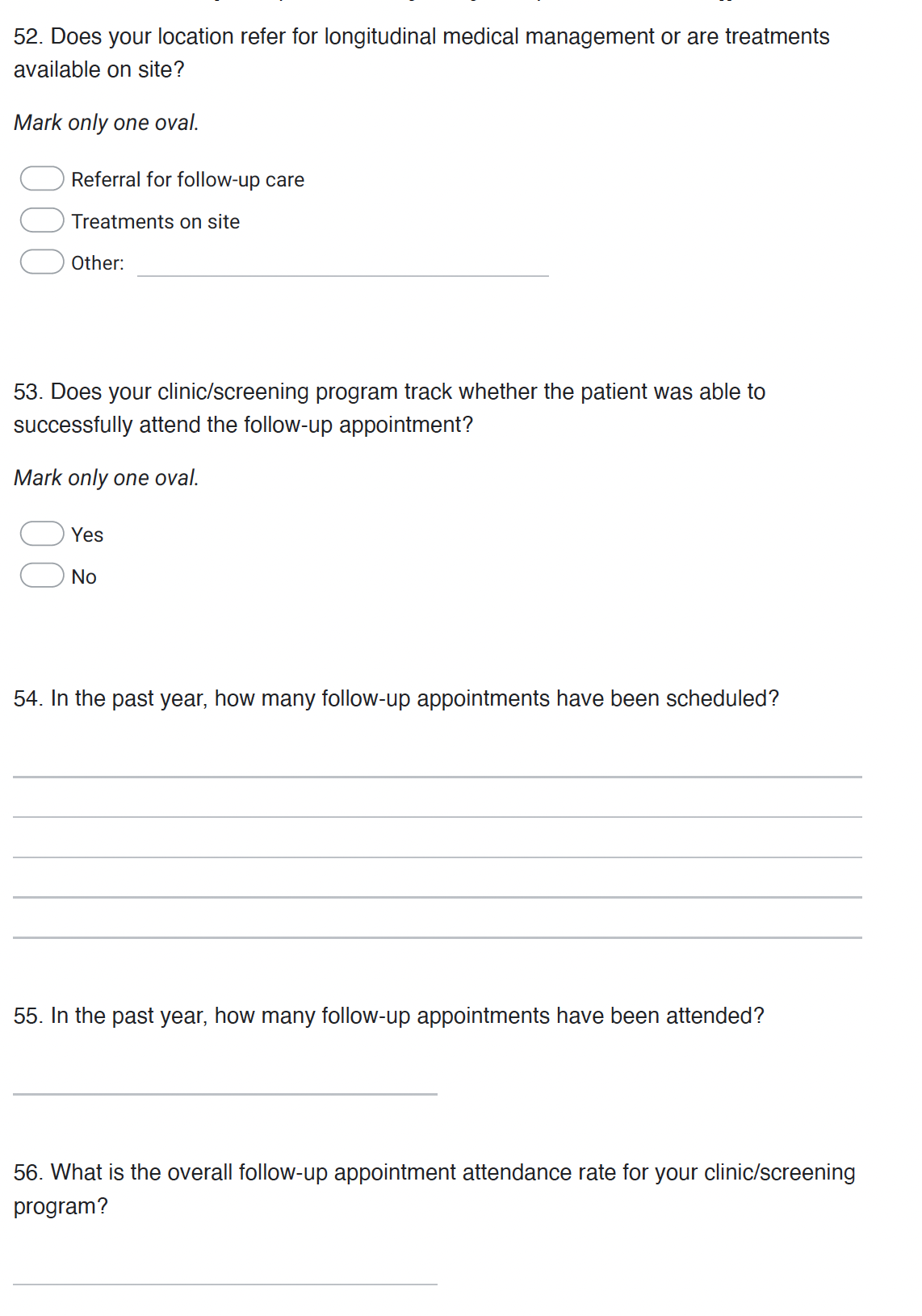


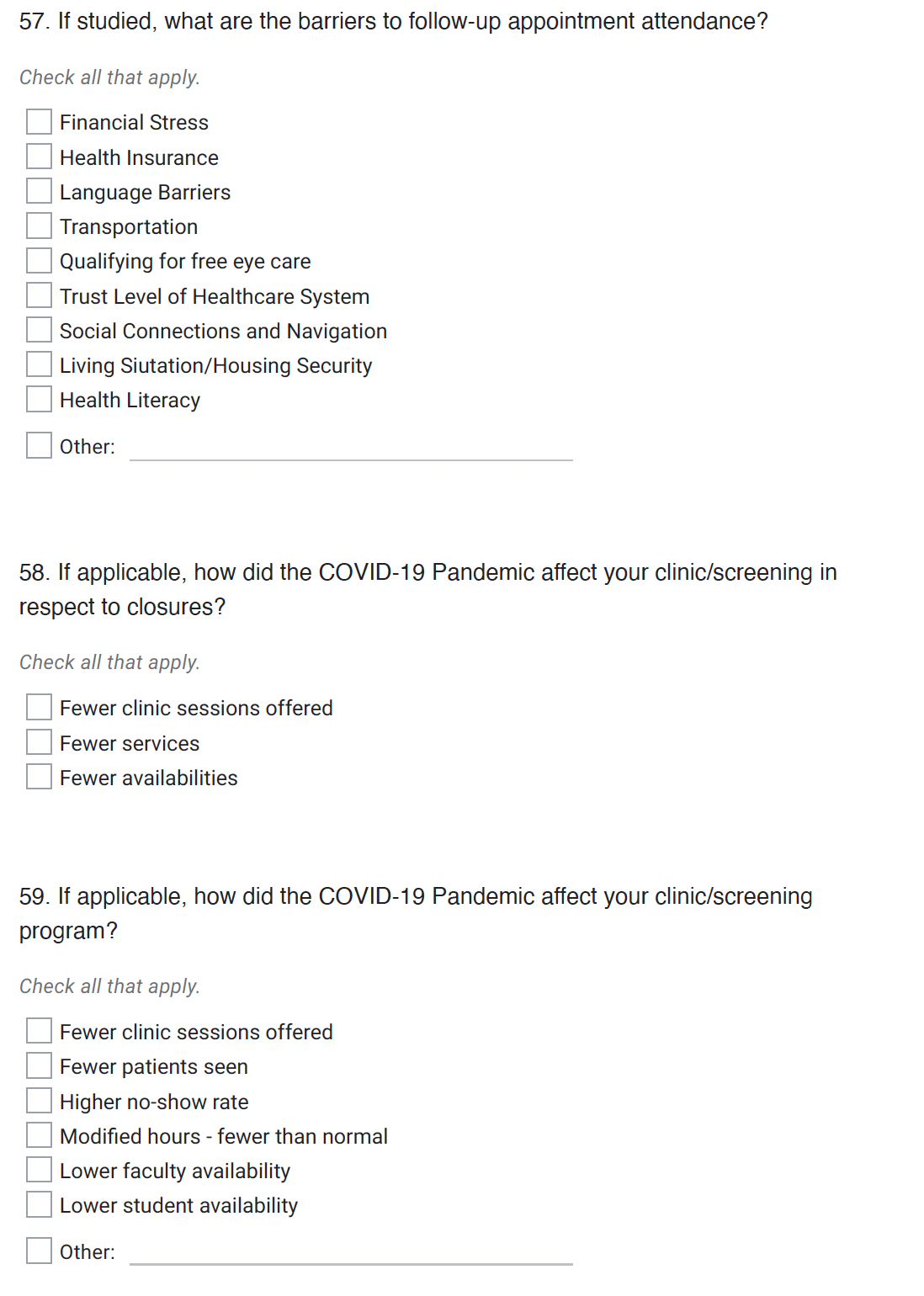


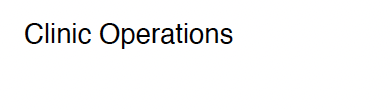


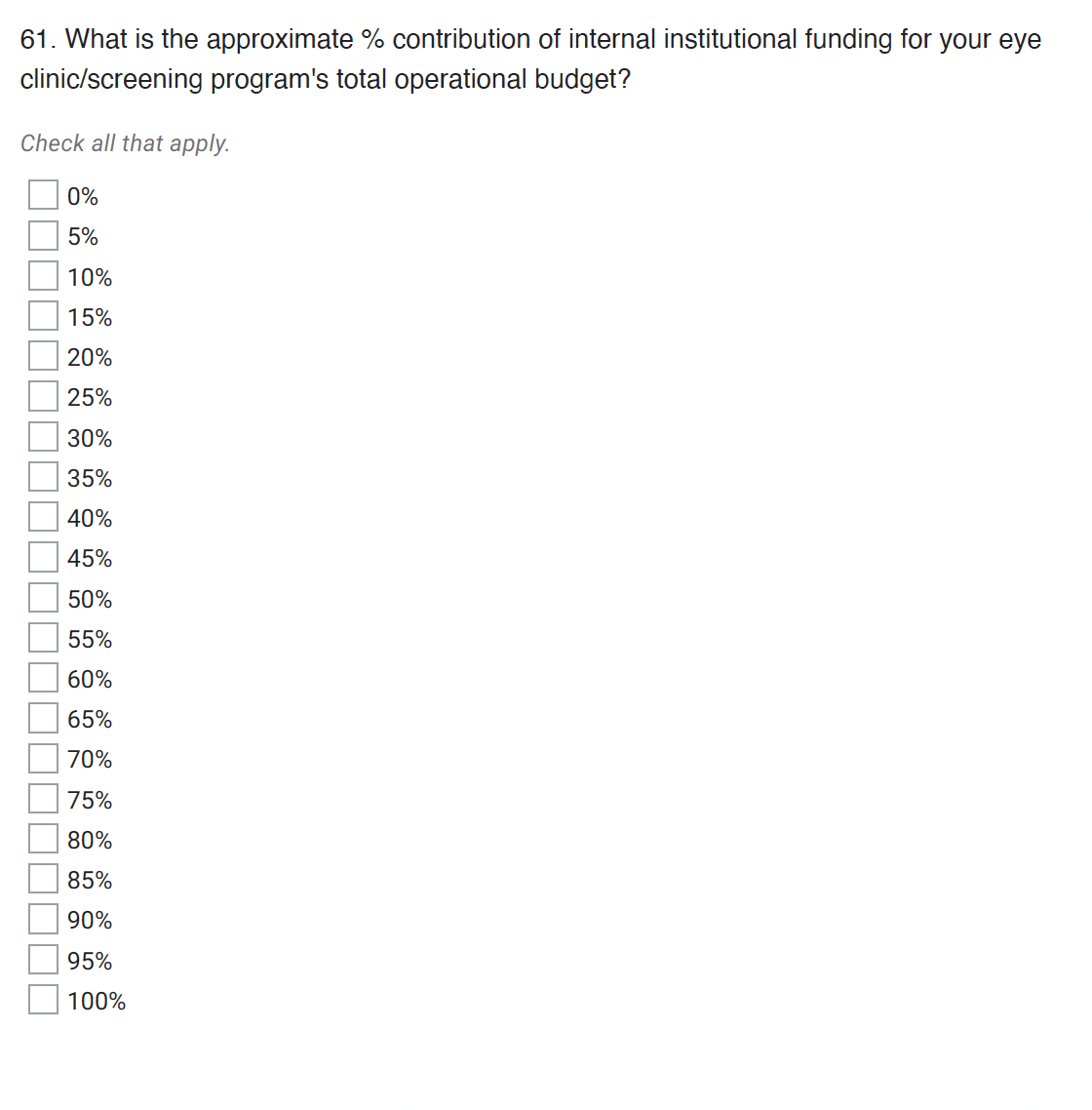


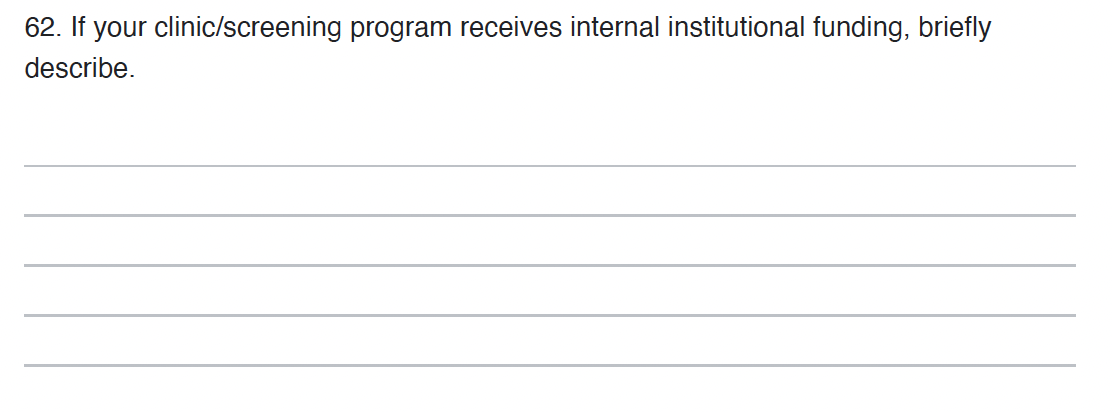


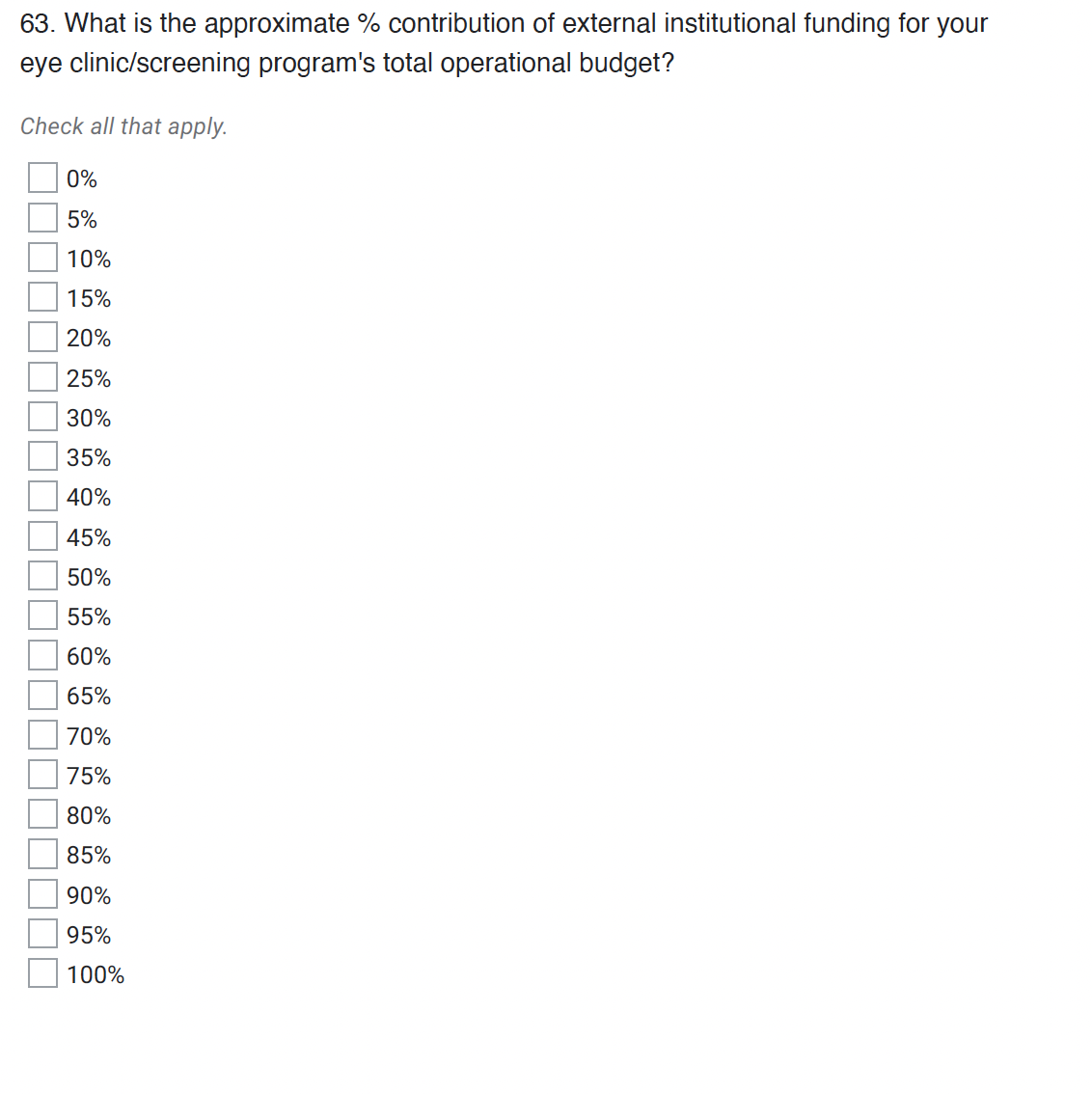


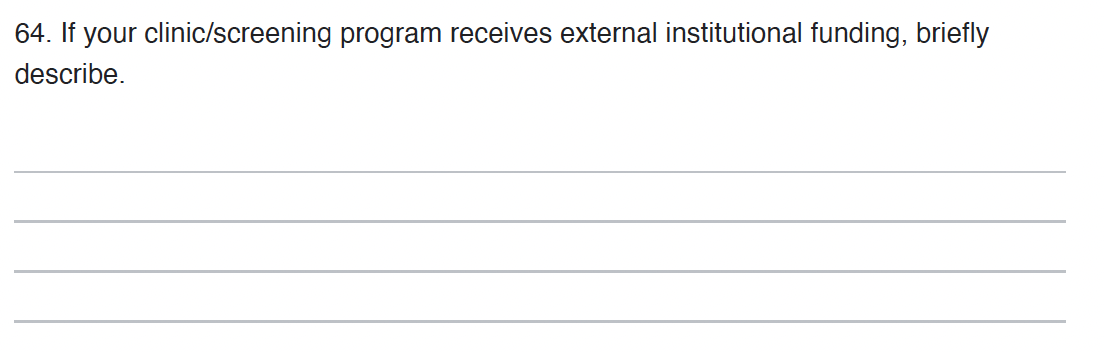


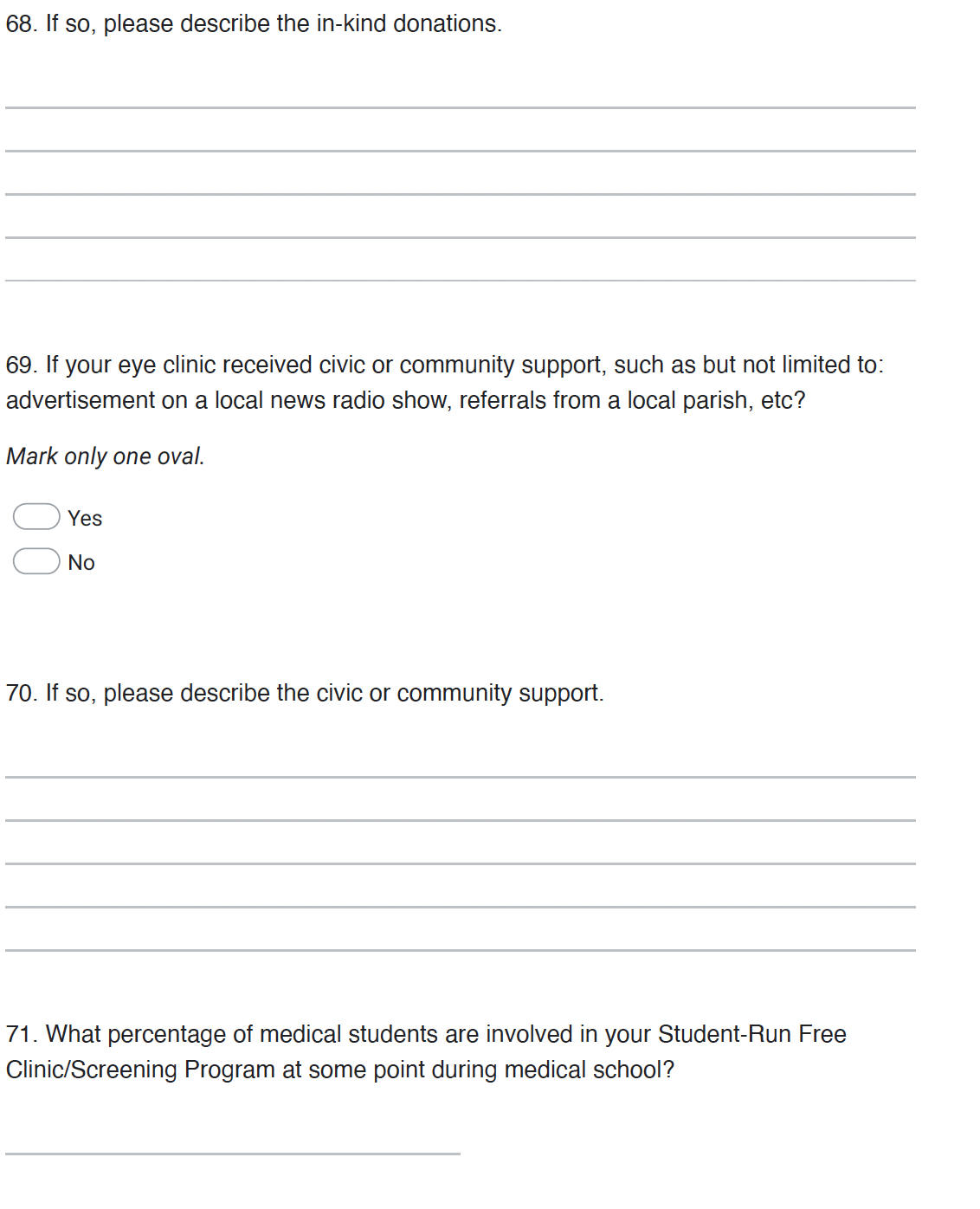

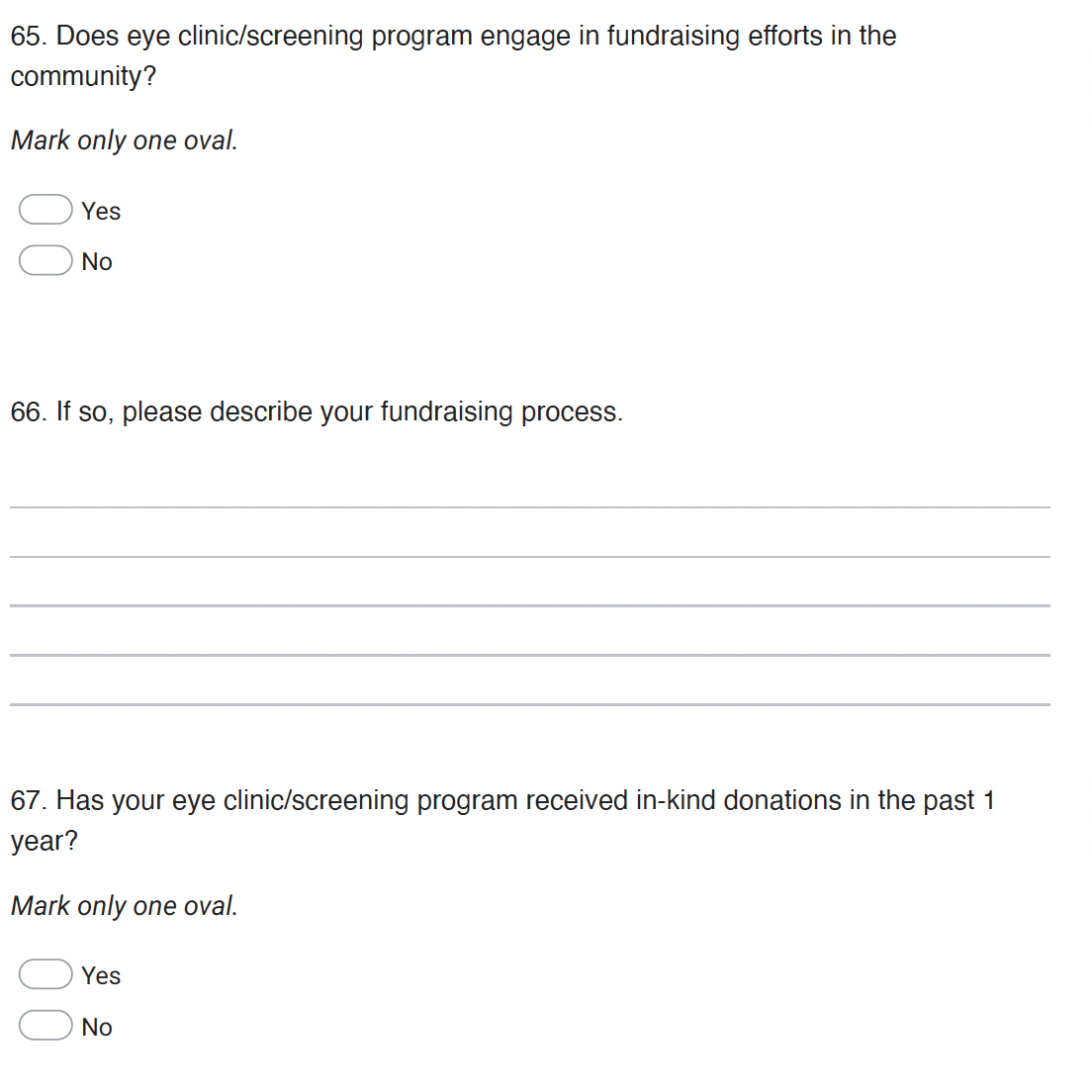


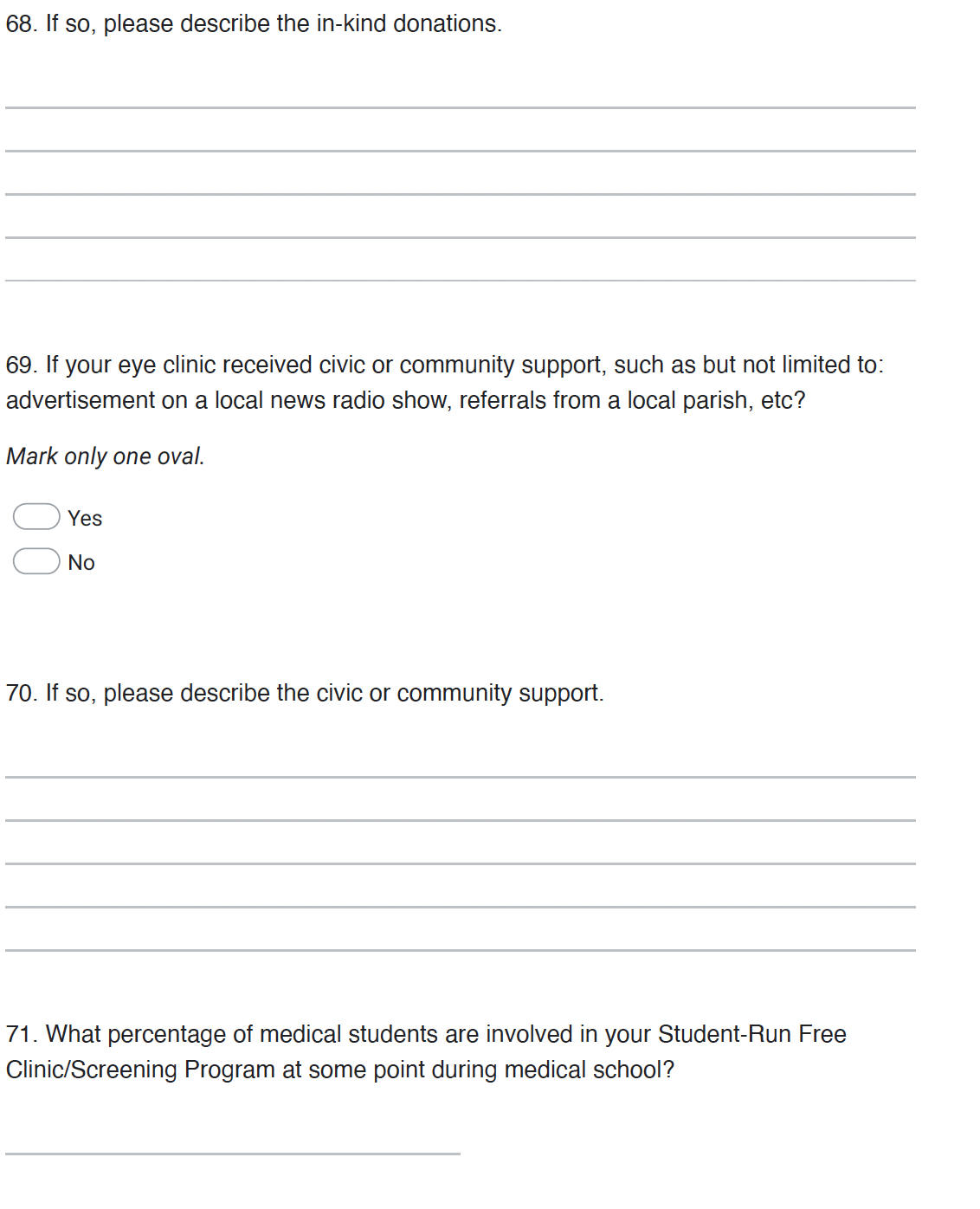


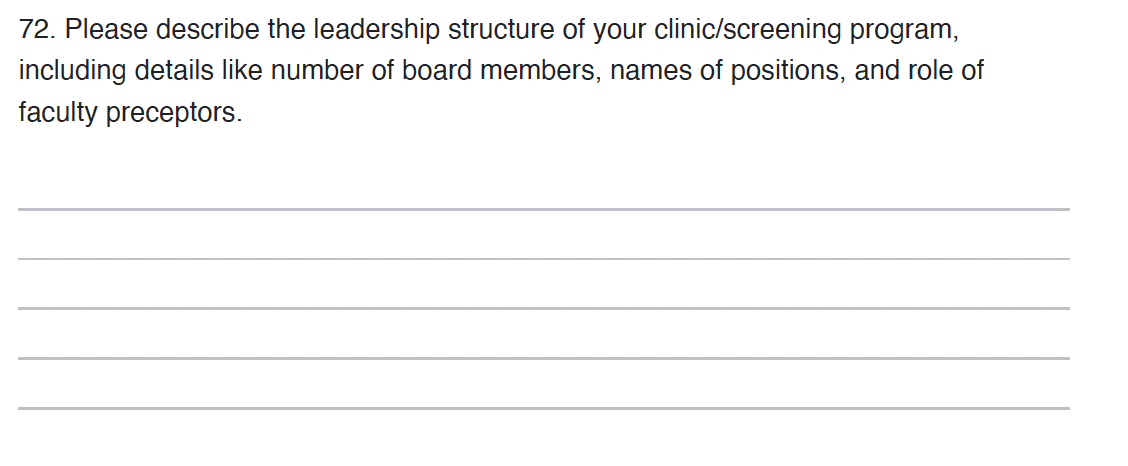


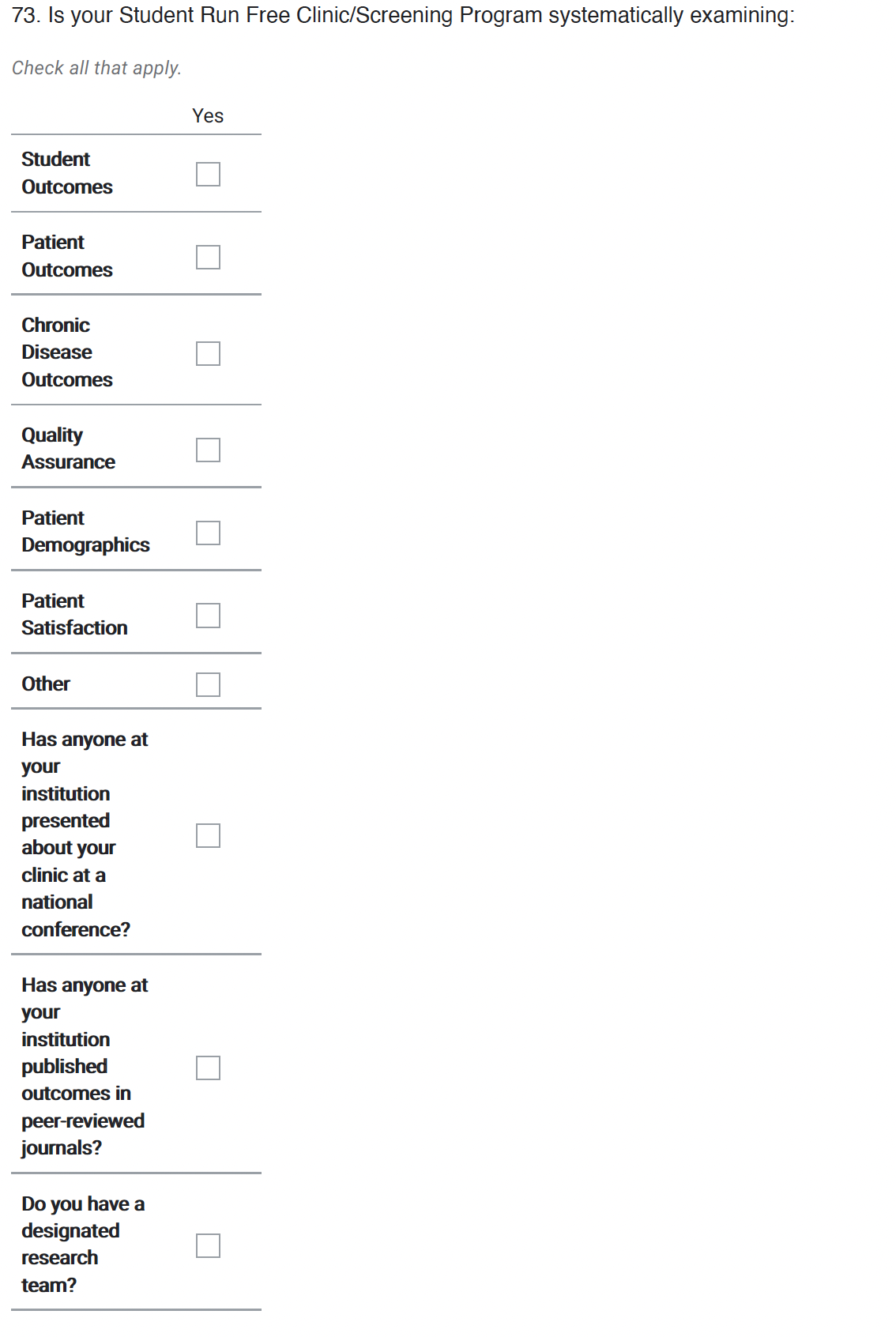


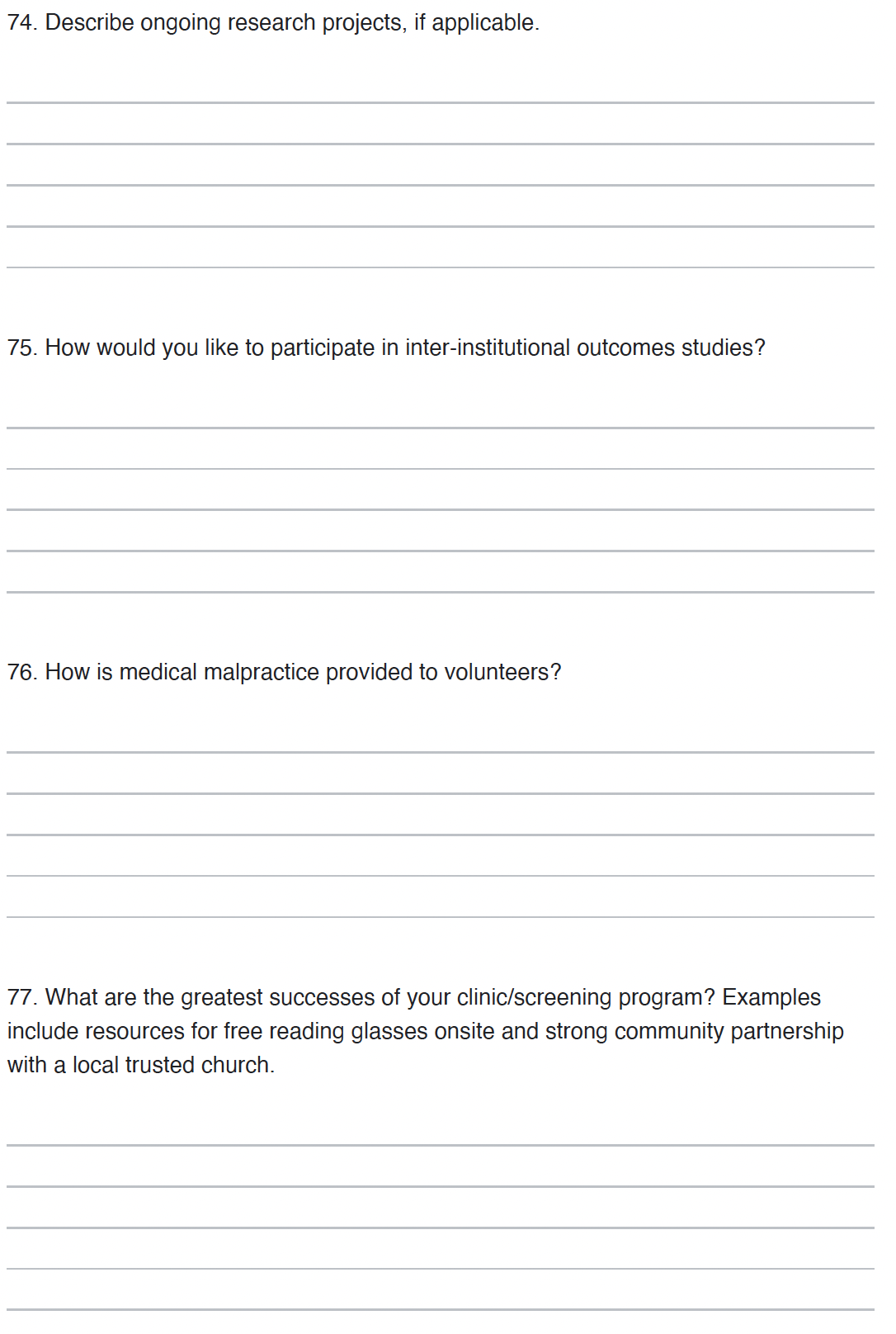


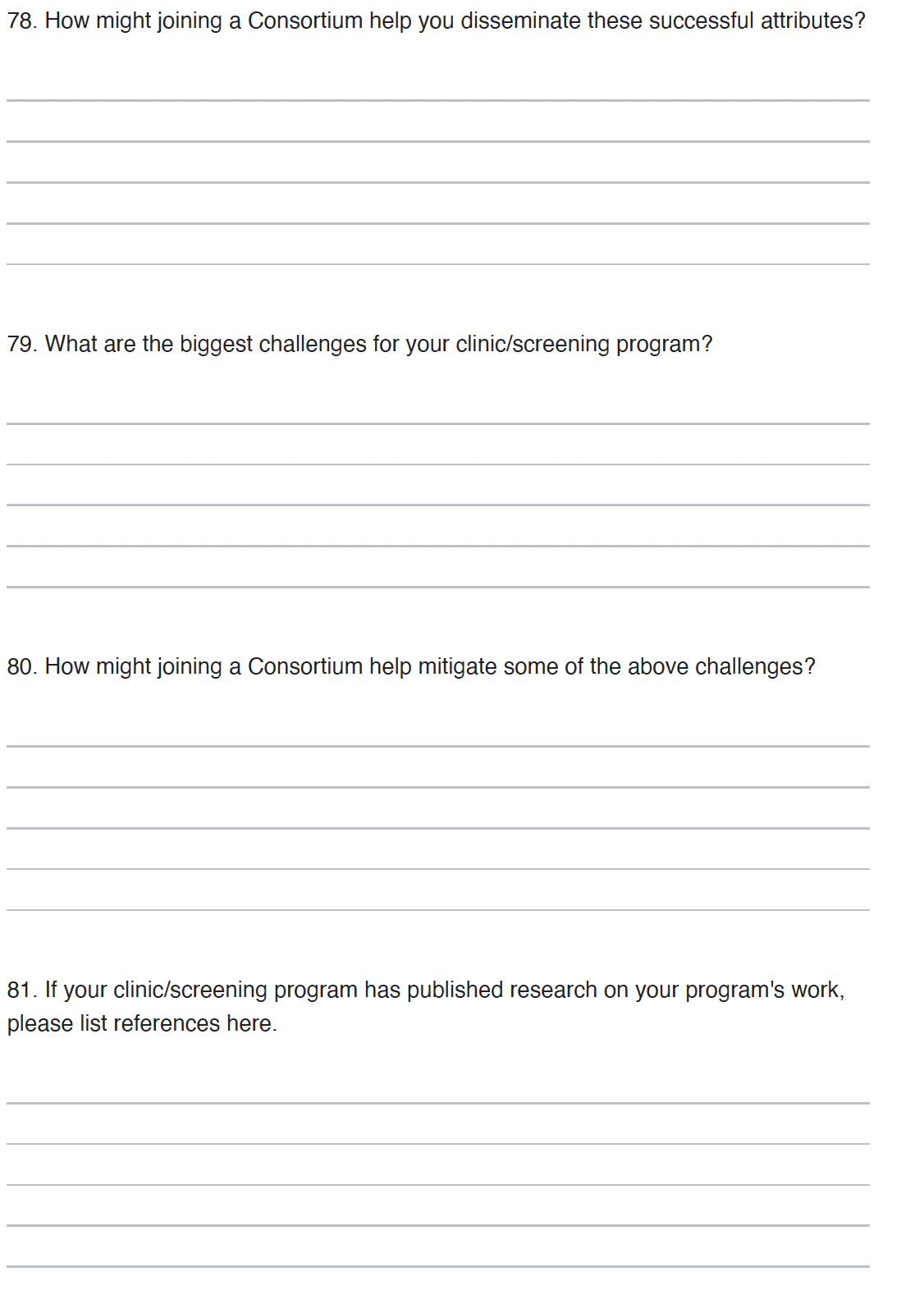

Supplement: Supplementary file 1 — Supplementary Material 1 [file 12909_2024_6396_MOESM1_ESM.docx]
